# The Role of RNA Splicing Factors in Cancer: Regulation of Viral and Human Gene Expression in Human Papillomavirus-Related Cervical Cancer

**DOI:** 10.3389/fcell.2020.00474

**Published:** 2020-06-12

**Authors:** Andrea Cerasuolo, Luigi Buonaguro, Franco M. Buonaguro, Maria Lina Tornesello

**Affiliations:** Molecular Biology and Viral Oncology Unit, Istituto Nazionale Tumouri IRCCS–Fondazione G. Pascale, Naples, Italy

**Keywords:** splicing factors, cervical cancer, human papillomavirus (HPV), RNA, heterogeneous nuclear ribonucleoproteins (hnRNPs), serine/arginine-rich proteins (SR)

## Abstract

The spliceosomal complex components, together with the heterogeneous nuclear ribonucleoproteins (hnRNPs) and serine/arginine-rich (SR) proteins, regulate the process of constitutive and alternative splicing, the latter leading to the production of mRNA isoforms coding multiple proteins from a single pre-mRNA molecule. The expression of splicing factors is frequently deregulated in different cancer types causing the generation of oncogenic proteins involved in cancer hallmarks. Cervical cancer is caused by persistent infection with oncogenic human papillomaviruses (HPVs) and constitutive expression of viral oncogenes. The aberrant activity of hnRNPs and SR proteins in cervical neoplasia has been shown to trigger the production of oncoproteins through the processing of pre-mRNA transcripts either derived from human genes or HPV genomes. Indeed, hnRNP and SR splicing factors have been shown to regulate the production of viral oncoprotein isoforms necessary for the completion of viral life cycle and for cell transformation. Target-therapy strategies against hnRNPs and SR proteins, causing simultaneous reduction of oncogenic factors and inhibition of HPV replication, are under development. In this review, we describe the current knowledge of the functional link between RNA splicing factors and deregulated cellular as well as viral RNA maturation in cervical cancer and the opportunity of new therapeutic strategies.

## Introduction

The large majority of human genes are transcribed as pre-mRNAs, containing non-coding (introns) and coding sequences (exons), that are processed by spliceosomal complexes to remove introns and produce mature mRNAs (Shi, 2017). The alternative removal of introns from pre-mRNAs and joining of exons into different mature transcripts enables the translation of multiple proteins from the transcription of a single gene (Bush et al., 2017). For this reason, the approximately 20,000 human genes are able to encode at least 100,000 different proteins (Wang et al., 2015).

Cell proteins generated by alternative splicing are selectively expressed in a tissue-specific and time-dependent manner and contribute to the regulation of numerous metabolic pathways involved in cell cycle control, differentiation and apoptosis (Baralle and Giudice, 2017). Aberrant splicing may cause the production of abnormal mRNA isoforms encoding mutated proteins with gain or loss of functions that are involved in neoplastic cell transformation, cancer development and metastasis (Oltean and Bates, 2014).

The role of spliceosome complexes and splicing regulatory factors in cancer has been widely investigated (Oltean and Bates, 2014). In particular, the snRNPs, the hnRNPs and the SR proteins have been shown to act either as oncoproteins or tumor suppressor proteins in different cancer types, including cervical neoplasia (Sorlie et al., 2001; Dvinge et al., 2016; Kohler et al., 2016; Cheng et al., 2017).

The aim of this review was to summarize the current studies on the role played by splicing factors in different cancer types with a particular focus on their peculiar activity in HPV-related cervical neoplasia. Indeed, in HPV infected cells the splicing factors are able to modulate the maturation either of cellular transcripts or of viral RNAs leading to the viral life cycle completion or production of viral and host cell transcripts encoding oncoproteins that cause transformation of cervical epithelium.

## RNA Splicing Factors

The removal of introns from pre-mRNAs is a process catalyzed by two large ribonucleoprotein complexes, namely major and minor spliceosomes, in cooperation with numerous splicing factors (Grabowski et al., 1985; Papasaikas and Valcarcel, 2016). The major spliceosome, responsible for more than 99% of splicing reactions in human cells, is composed of five uridine-rich small nuclear RNAs (snRNA U1, U2, U4, U5, and U6) and over 100 snRNA associated proteins (snRNPs) that undergo complex conformational changes during the different phases of splicing reactions (Frendewey and Keller, 1985; Wahl et al., 2009; Will and Luhrmann, 2011; Kastner et al., 2019). On the other hand, the minor spliceosome, including the U5 snRNA as well as functional analogs of the major spliceosome snRNAs (U11, U12, U4atac and U6atac), catalyzes the splicing of the less abundant U12-type introns (Tarn and Steitz, 1997; Turunen et al., 2013).

**FIGURE 1:**
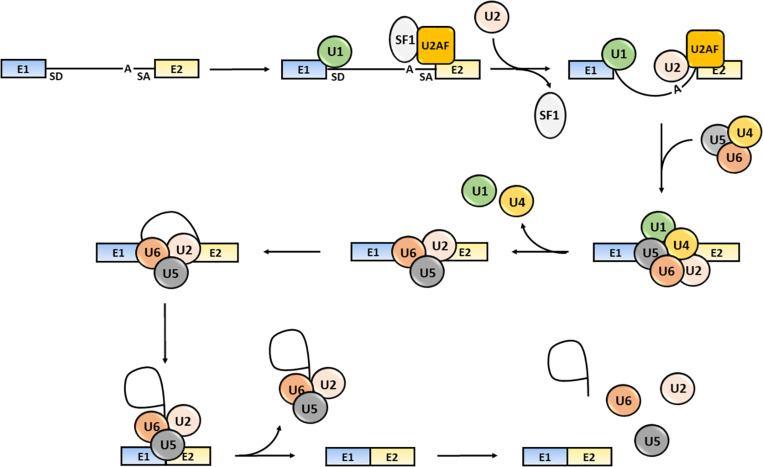
Steps of the splicing process. The snRNP U1, SF1 and U2AF bind to the intron 5′-end SD site, to an intronic branch point site (A) and to the intron 3′-end SA site, respectively. The U2 displaces SF1 and the U4/U5/U6 snRNPs trimer interacts with snRNPs U1 and U2 causing U1 and U4 release. The activated spliceosome catalyzes the cleavage of the intron 5′-end, the formation of a lariat, the intron the cleavage of the intron 3′-end, the release of the lariat/U2/U5/U6 complex release and joining of exons (Shi, 2017). Exons are defined as “E”; the intron is represented as a black solid line.

The splicing process consists of several sequential reactions involving recruitment of spliceosome components and interaction with *cis*-acting regulatory intronic sequences, such as the 5′ splice donor (SD) and 3′ splice acceptor (SA) sites, the intervening branch-point and the polypyrimidine tract (Aebi et al., 1986; Wang and Burge, 2008). Briefly, the formation of an early spliceosome complex involves the interaction of the U1 snRNP with the 5′ splice site through the base pairing of the U1 snRNA component, the binding of the U2 accessory factor (U2AF) to the polypyrimidine region and the connection of U2AF to U1 through the bridging splicing factor SF1. Binding of U2 snRNA to the branch sequence and 3′ splice site facilitates the U1 and U2 snRNPs interaction and the formation of the spliceosome complex A. Then, the U4/U5/U6 snRNP trimer interacts with the U1 and U2 snRNPs forming the spliceosome complex B, which releases the U1 and U4 snRNPs and becomes activated (complex C) (Wahl et al., 2009). The active spliceosome causes the final exclusion of the intron through: (1) the cleavage of the intron 5′-end; (2) the formation of a lariat (the 5′-end of the intron binds to the branch point); (3) the cleavage of the intron 3′-end; (4) the release of the lariat/U2/U5/U6 complex; and (5) the joining of exons (Lee and Rio, 2015; Papasaikas and Valcarcel, 2016) (Figure 1).

**FIGURE 2:**
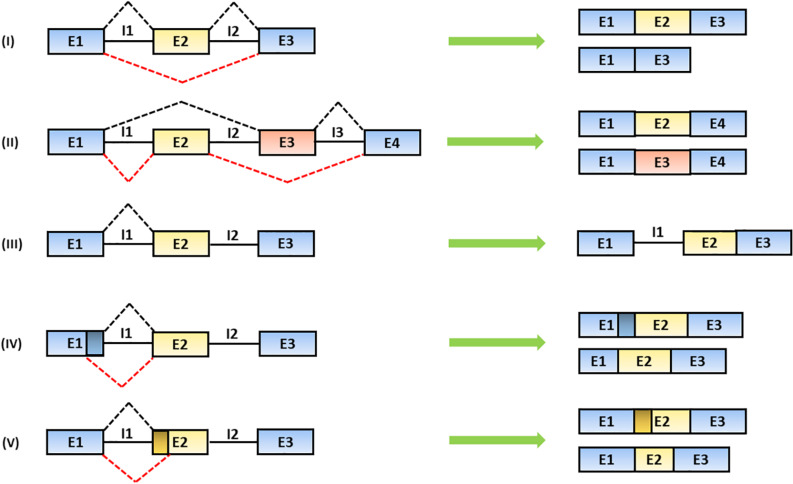
Mechanisms of alternative splicing. The spliceosome generates distinct mRNA isoforms by alternative usage of splice donor (SD) and acceptor (SA) sites, located at the 5′ and 3′ end of introns, respectively. The splicing products include: (I) exon skipping (the partial or total removal of exons), (II) inclusion of mutually exclusive exons, (III) intron retention and the use of alternative (IV) SD or (V) SA sites (Baralle and Giudice, 2017). In the figure, black solid lines represent introns; black and red dashed lines represent alternative splicing mechanisms. Exons are indicated as “E” and introns are indicated as “I.”

The alternative recognition of differential splice donor and acceptor sites allows the production of several mRNA isoforms according to five different figures: (1) the exon is totally or partially skipped; (2) mutually exclusive exons are included; (3) introns are retained; (4) alternative splice donor; or (5) splice acceptor sites are selectively chosen leading to the production of exons with different lengths (Lee and Rio, 2015; Wang et al., 2015) (Figure 2).

**FIGURE 3:**
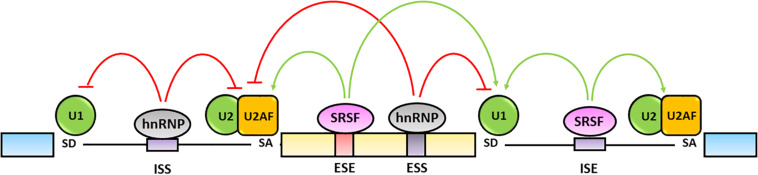
Regulation of alternative splicing. Alternative splicing is finely regulated by trans-acting hnRNPs and SRSFs protein families. The hnRNPs generally bind exonic (ESSs) and intronic splicing silencers (ISSs), antagonize SRs activity and inhibit spliceosome assembly on the splicing sites (red lines). The SRSFs bind to exonic (ESEs) and intronic (ISEs) splicing enhancers and increase the splicing efficiency by favoring the spliceosome recruitment and assembly on the splicing sites (green lines) (Chen and Manley, 2009; Wang et al., 2015).

The fine tuning of pre-mRNA maturation is regulated by *trans*-acting splicing factors including hnRNPs, that generally inhibit the splicing by binding to exonic (ESSs) or intronic splicing silencers (ISSs) sequences, and SR proteins that typically activate the splicing by interacting with exonic (ESEs) and intronic (ISEs) splicing enhancers (Wang and Burge, 2008; Chen and Manley, 2009) (Figure 3).

The hnRNPs are a protein family comprising 20 major RNA-binding proteins that contain a RRM, a quasi-RRM domain and a K Homology (KH) domain binding to pre-mRNA sequences as well as a glycine-rich domain interacting with other hnRNPs (Busch and Hertel, 2012). The canonical function of hnRNPs is the inhibition of splicing reactions through their binding to cognate sites and repression of either the assembly of the spliceosome complex on the 5′-SD and 3′-SA sites or the recruitment of SR proteins on ESEs and ISEs following multimerization along exons or by looping out entire exons (Nasim et al., 2002; Geuens et al., 2016; Dvinge, 2018; Ule and Blencowe, 2019) (Figure 3). On the other hand, some hnRNPs have been shown to promote splicing through their interaction with ISE sequences containing G triplets. Indeed, hnRNP A1 and hnRNP F have been recognized to bind a AGGGA sequence in the 5′ GA-rich enhancer within the intron 10 of the insulin receptor gene (INSR) transcript as well as similar motifs located at the 3′ end of the same intron and to regulate the skipping or the inclusion of exon 11 causing the differential expression of the insulin receptor A or B isoforms, respectively (Talukdar et al., 2011).

Most of the hnRNPs, including hnRNP A1/A2, hnRNP B1/B2, hnRNP E, hnRNP J, and hnRNP K, are localized to the nucleus and after homomeric and heteromeric complexes formation they shuttle to the cytoplasm via the transportins binding (Siomi and Dreyfuss, 1995; Mili et al., 2001; Geuens et al., 2016). On the other hand, hnRNP C and hnRNP U possess a nuclear retention sequence that inhibits transfer to the cytoplasm (Nakielny and Dreyfuss, 1996; Mili et al., 2001). Post-translational modifications of hnRNPs, such as serine and threonine phosphorylation, arginines methylation, SUMOylation and ubiquitination, affect their sub-cellular localization and modulate positively or negatively their activity (Lee et al., 2012; Friend et al., 2013; Moujalled et al., 2015; Wang et al., 2017). For instance, the ERK kinase was shown to phosphorylate hnRNP K serines 284 and 353 in response to stress stimuli and to cause cytoplasmic accumulation and inhibition of hnRNP K-mediated translation in HeLa cells (Habelhah et al., 2001). In addition, the hnRNPs are able to modulate also the mRNA stability, capping, transport and poly-adenylation as well as to regulate the telomeres maintenance and chromatin organization (Han et al., 2010; Geuens et al., 2016).

The SR proteins family includes twelve serine-arginine rich splicing factors (SRSF1 to 12) characterized by canonical RRM, that interact with the ESE and ISE elements, and C-terminal serine-arginine repeats, which facilitate the recruitment of the spliceosome components to the splice sites (i.e., the binding of U1 to the 5′-SD site) and promote splicing processes (Manley and Krainer, 2010; Shen and Mattox, 2012; Bradley et al., 2015; Jeong, 2017) (Figure 3). In a few cases the SR proteins have been shown to bind silencer elements and to repress splicing (Kanopka et al., 1996; Shin et al., 2004; Shen and Mattox, 2012). For example, SRSF1 (SF2/ASF), SRSF4 (SRp75), SRSF5 (SRp40), and SRSF6 (SRp55) are able to bind an ISS located in the intron 9 of the CFTR transcript and to skip the exon 9 causing the production of a nonfunctional protein associated with cystic fibrosis development (Pagani et al., 2000).

The localization of SR proteins is regulated by dynamic serine-arginine repeats phosphorylation/dephosphorylation cycles that also modify their protein–protein interaction and functional activities (Zhou and Fu, 2013; Long et al., 2019). For example, the SRSF1, SRSF3 (SRp20), and SRSF7 (9G8) phosphorylated proteins are bound and shuttled by transportin SR2 from the cytoplasm to the nucleus (Lai et al., 2000, 2001). The serine-arginine rich proteins are specifically phosphorylated by the serine/arginine-protein kinases (SRPKs) and the Cdc2-like kinase/Ser-Thr-Tyr (Clk/Sty) kinases (Colwill et al., 1996; Giannakouros et al., 2011). The SRPKs are constitutively active in normal cells and localize either in the cytoplasm or into the nucleus (Giannakouros et al., 2011). The nuclear kinase CLK1 has been shown to phosphorylate the RS2 domain of SRSF1 and to cause the re-localization from speckles to the nucleoplasm (Aubol et al., 2018). In particular conditions, the SR proteins are phosphorylated by other kinases such as topoisomerase 1 (TOPO1), which becomes active in response to extra-cellular stimuli, and by Akt kinase and MAP kinases (MAPKs) that are constitutively active in cancer cells (Rossi et al., 1996; Naro and Sette, 2013). The SRSFs, similarly to hnRNPs, have also a variety of non-canonical activities related to the stability, export and translation of mRNAs as well as to the chromatin remodeling, nucleolar stress response, genome stability and cell cycle regulation (Howard and Sanford, 2015).

## Deregulation of the Splicing Machinery in Cancer

Several studies demonstrated that deregulation of constitutive and alternative splicing plays a crucial role in carcinogenesis and could be considered a novel cancer hallmark (Oltean and Bates, 2014; El and Younis, 2018; Wang and Lee, 2018; Zhang Y. et al., 2019; Che and Fu, 2020). Indeed, recent transcriptome sequencing analyses demonstrated that splicing factors expression is commonly deregulated in a multitude of cancer types and is associated with alterations of the tissue-specific transcripts as well as modification of protein–protein interactions (Buljan et al., 2012; Singh and Eyras, 2017).

Characterization of known transcripts from RNA-seq data of 4,542 tumor samples from 11 cancer types recorded in TCGA revealed the presence of 8,122 switches in the RNA isoforms encoded by 6,442 genes, the most common being RAC1 gaining an extra Ras domain and TP53 losing coding capacity (Climente-Gonzalez et al., 2017). Such changes were found mainly associated with alterations in apoptosis, ubiquitin-mediated proteolysis, ERBB-signaling, RNA transcription and splicing pathways (Climente-Gonzalez et al., 2017). In addition, the comprehensive analysis of whole exome sequencing and RNA-seq data of 8,705 tumor samples and 640 normal control tissues from 32 cancer types, including cervical cancer, showed approximately 251,000 new exon–exon junctions in tumors (around 930 per sample), of which 18,000 were recurrent events detected in at least 100 samples (Kahles et al., 2018). Such novel exon–exon junctions were predicted to produce “neoantigens” and to affect the immune response (Kahles et al., 2018). David et al. (2020) extended the analysis of exon–exon junctions to 10,549 tumor samples across 33 TCGA cancer types and observed that more than 50% of new junctions were not shared among cancer samples. Importantly, in cervical cancer they identified a total of 14,086,434 exon–exon junctions of which 2,263,326 were specific to cervical cancer (David et al., 2020).

Several mechanisms are responsible for aberrant splicing processes in human cancers. First, the uncontrolled overexpression of splicing factors may cause anomalous splicing events in tumors (Sveen et al., 2016). The up-regulation of hnRNPs and SRSFs has shown to be commonly caused by gene rearrangements and copy number variations in a variety of cancer types (Shilo et al., 2015). For example, the HNRNPA2B1 gene was found amplified in glioblastoma and its copy number was demonstrated to be inversely correlated with patients survival (Golan-Gerstl et al., 2011). Moreover, the amplification of the chromosome 17q23 and chromosome 6p21 regions has been shown to cause the overexpression of SRSF1 in breast cancer and SRSF3 in lung and cervical carcinoma, respectively (Parssinen et al., 2007; Jia et al., 2010).

The expression of hnRNP A1, hnRNP A2 and hnRNP I (also known as polypyrimidine tract binding protein, PTB) was demonstrated to be trans-activated by Myc oncoprotein. Specifically, the Myc mediated up-regulation of hnRNP A1, hnRNP A2 and hnRNP I induces the production of the pyruvate kinase embryonic isoform PKM2 and the activation of the aerobic glycolysis in different cancer cells (Christofk et al., 2008; David et al., 2010; Chen H. et al., 2018). Myc was also demonstrated to bind E-boxes in the SRSF1 promoter and to transactivate the SRSF1 overexpression that cause production of MKNK2 +13B and TEAD1 +5 isoforms and enhanced proliferation of lung cancer cells (Das et al., 2012).

Altered processing of splicing regulator transcripts represents a further mechanism of abnormal pre-mRNA maturation (Czubaty and Piekielko-Witkowska, 2017). For instance, knockdown experiments of S6K2 kinase, that phosphorylates the serine^6^ residue in hnRNP A1 protein, caused enhanced production of the PKM2 isoform and increased glycolysis in colorectal cancer cells (Sun Y. et al., 2017). The overexpression of SRPK1 causes the hyper-phosphorylation of SRSF1, leading to increased production of Rac1b, an oncogenic variant of the GTPase Rac1 signaling protein that promotes survival and proliferation of colorectal cancer cells (Goncalves et al., 2014; Patel et al., 2019). The overexpression of Clk2 kinase was also shown to sustain cell growth in breast cancer, while its silencing was demonstrated to reduce the SRSF1 phosphorylation and to induce the production of the ENAH isoform, which is typical of the mesenchymal phenotype and associated with tumor invasion and metastasis (Yoshida et al., 2015). *In vitro* studies demonstrated that the up-regulation of the NEK2 kinase promotes the production of the anti-apoptotic BCL-X_L_ variant through the anomalous phosphorylation of SRSF1 (Naro et al., 2014). In addition, the PI3K/Akt signaling pathway, constitutively activated by mutated EGFR, leads to SRSF1 hyper-phosphorylation and consequent reduction of the anti-apoptotic isoform Casp-9b in lung cancer (Shultz et al., 2010).

Other post-translational modifications, such as acetylation and ubiquitination, are important modulators of splicing factors activity. For example, the hyper-acetylation of hnRNP A1 induced by high glucose levels in hepatocellular carcinoma cells promotes the production of the PKM2 variant and consequent enhancement of glucose metabolism as well as transcription of genes responsible for cell proliferation and growth, such as GLUT1, LDHA, PDK1, CCND1, and MYC (Yang et al., 2019). In addition, the hyper-acetylation of SRSF5 by Tip60 acetyl-transferase causes the production of the CCAR1 isoform S, which favors tumor growth by promoting glucose consumption and acetyl-CoA production (Chen et al., 2018a). On the other hand, the hypo-acetylation and consequent degradation of SRSF5 by the Smurf1 ubiquitin ligase causes CCAR1S reduction under low intracellular glucose levels (Chen et al., 2018a).

The hyper-*O*-GlcNAcylation of hnRNP-K in cholangiocarcinoma cells induces its translocation to the nucleus, where it drives the expression of genes involved in the extracellular matrix composition, cell movement, angiogenesis and epithelial mesenchymal transition, such as CCK, MMP3, PTGS2, and CTGF as well as CCND1 and XIAP (Gao R. et al., 2013; Phoomak et al., 2019). The overexpression of the SUMO1 was shown to cause the sumoylation of hnRNP K and increased *c-Myc* transcription associated with cell proliferation in Burkitt lymphoma (Suk et al., 2015).

Recurrent somatic mutations in splicing factors encoding genes have also been shown to affect the splicing processes in cancer (Kandoth et al., 2013; Watson et al., 2013; Sveen et al., 2016). Specifically, oncogenic driver mutations have been identified in 119 genes encoding for splicing factors in a variety of tumors (Seiler et al., 2018). For instance, point mutations and deletions in the HNRNPK gene, causing hnRNP K down-regulation, have been suggested to have a role in the development of acute myeloid leukemia (Sweetser et al., 2005; Gallardo et al., 2015). Moreover, hotspot mutations in the SRSF2 gene, such as P95H, change the binding properties of the SRSF2 protein and cause genome-wide splicing network alteration and aberrant maturation of different hnRNPs, including HNRNPA2B1, HNRNPM, HNRNPH1, and HNRNPH3, especially in myelodysplastic syndromes (Komeno et al., 2015; Arbab et al., 2018; Aujla et al., 2018; Liang et al., 2018; Masaki et al., 2019).

Moreover, nucleotide changes within the exonic or intronic *cis*-acting splicing factors binding motifs may result in the disruption or creation of binding sites and aberrant splicing reactions (Wang and Cooper, 2007; Sterne-Weiler and Sanford, 2014). A recent whole-exome and transcriptome comprehensive study including 31 cancer types identified 14,438 splicing-associated variants, most of which were shown to disrupt donor and acceptor sites and to cause exon skipping, intron retention and alternative 5′ or 3′ splicing site usage particularly in TP53 and GATA3 genes (Shiraishi et al., 2018).

Additionally, non-coding RNAs, such as miRNAs, are able to regulate splicing and transcription both in normal and tumor tissues for their ability to target the 3′-UTR of hnRNPs and SRSFs mRNAs (Anczukow and Krainer, 2016). For instance, the miR-15a-5p and miR-25-3p have been described as negative regulators of hnRNP A1 expression, required for the maturation of miR-18a-3p, that in turn inhibits the K-RAS oncogene in ovarian cancer (Rodriguez-Aguayo et al., 2017). Besides, miR-183-5p and miR-200c-3p in renal cancer as well as miR-193a-3p in gastric cancer have been demonstrated to target the SRSF2 (SC35) 3′-UTR mRNA, affecting the maturation of several pre-mRNAs involved in the apoptosis (Sokol et al., 2018; Lee et al., 2019).

On the other hand, the hnRNP and SRSF proteins are able to control directly or indirectly the expression of several miRNAs and other non-coding RNAs involved in cancer development (Ratnadiwakara et al., 2018). Indeed, the hnRNP A1 has been shown to interact with pri-miR-18a conserved terminal loop and to induce a relaxation in the stem loop that facilitates the cleavage by Drosha and production of mature miR-18a in prostate, esophageal, pancreatic, hepatocellular, and colorectal cancer (Guil and Caceres, 2007; Komatsu et al., 2014; Kooshapur et al., 2018). Moreover, the hnRNP D reduces the Dicer1 levels by targeting the 3′-UTR of DICER1 mRNA, causing down-regulation of tumor suppressor miR-122 and increased viability of PLC/PRF/5 hepatoma as well as Huh7 liver derived cell lines (Wu et al., 2018). Among SRSF proteins, the SRSF3 was demonstrated to enhance the processing of miR-16-1, miR-30a and miR-223 through the binding of a CNNC motif located 17-18 nucleotides downstream the Drosha cleavage signal (Auyeung et al., 2013).

Several hnRNPs, especially hnRNP K, are also able to modulate the expression of genes involved in the carcinogenic processes through their interaction with lncRNAs (Sun X. et al., 2017). In particular, Li et al. (2018) observed that linc00460 was overexpressed in non-small cell lung tumors and was able to interact with hnRNP K promoting migration and invasion of H460 and A549 human lung cancer cell lines. Furthermore, the analysis of RNA binding proteins-lncRNAs interaction network in the POSTAR2 database and co-immunoprecipitation experiments showed that SRSF1 was able to interact with and to stabilize the lncRNA NEAT1 causing increased cell proliferation in U87MG glioma cell line (Zhou et al., 2019).

Recently, hnRNPs have also been demonstrated to modulate the production of circular RNAs (Kramer et al., 2015). Specifically, the profiling of circular RNAs transcriptome in LNCaP prostate cancer cells expressing HNRNPL compared to HNRNPL-knockdown cells revealed the differential production of circ-PRKAR1B, circ-ZMIZ1, circ-FOXJ3, and circ-CCNY that have been suggested to be involved in prostate cancer development (Fei et al., 2017).

### Oncogenic Functions of hnRNPs and SRSFs

Several hnRNP and SRSF proteins have been demonstrated to be overexpressed in tumors and to possess multiple oncogenic functions (El and Younis, 2018) (Table 1). In particular, hnRNP A1 is upregulated in lung cancer and its silencing in lung cancer cell line A549 causes cell cycle arrest in G0/G1 phase (Liu et al., 2016). The hnRNP A1 expression enhances the production of a cyclin dependent kinase 2 (CDK2) isoform, characterized by the exon 5 retention, that promotes cell growth, while its inhibition causes the cell cycle arrest at the G2/M phase in oral squamous cancer cell lines (Yu et al., 2015). The overexpression of hnRNP A1 in gastric cancer and hnRNP A2/B1 in lung cancer has been shown to enhance the metastatic potential of tumor cells by inducing a shift from the expression of epithelial markers (i.e., E-cadherin) to mesenchymal markers (i.e., vimentin and snail) phenotype (Boukakis et al., 2010; Tauler et al., 2010; Chen et al., 2018b). The hnRNP A1 also mediates the production of the CD44v6 isoform, associated with larger tumor size, microvascular invasion and tumor recurrence in hepatocellular carcinoma patients (Zhou et al., 2013).

**TABLE 1:** Splicing factors and regulated oncogenic processes: physiological functions and oncogenic activities in different cancer types and cell lines.

Splicing factors (aliases)	Regulated processes	Oncogenic activities	Pathologic mechanism	Experimental model	Cancer types and cell lines	References
**hnRNPs**						
hnRNP A1	Splicing, mRNA export and stability, telomeres maintenance, translation	Induction of cell growth by regulation of CDK2 exon 5 alternative splicing	OE	KD	CAL 27 cell line	Yu et al. (2015)
hnRNP A2/B1	Splicing, mRNA localization and stability	Induction of EMT markers expression	OE	OE, KD	A549 cell line	Tauler et al. (2010)
hnRNP C1/C2	Splicing, mRNA transport and stabilization, translation	Maturation of miR-21, down-regulation of PDCD4, reduction of apoptosis, increase of proliferation and invasiveness	OE	KD	T98G cell line	Park et al. (2012)
hnRNP D	Telomeres maintenance, development, apoptosis, DNA recombination, mRNA decay	Up-regulation of GCH1, promoting cell proliferation and colony formation	OE	KD	Eca-109 cell line	Gao et al. (2016)
hnRNP E1	Splicing, mRNA stability, transcription, translation	Production of integrin β1A isoform promoting lymph node and hepatic metastases	OE	OE, KD	Pancreatic cancer	Jiang et al. (2017)
hnRNP E2	Splicing, mRNA stability, transcription, translation	Up-regulation of CDK2 stimulating cell proliferation	OE	KD	HGC-27 and MKN-45 cell lines	Chen C. et al. (2018)
hnRNP K	Splicing, transcription, translation, mRNA stability	Enhancement of cell migration and metastatization by up-regulation of MMP3, MMP10, PTGS2, ITGA6, CTGF, and RASA1	OE	OE	U2OS cell line	Gao R. et al. (2013)
hnRNP L	Splicing, mRNA export and stability, riboswitch	Activation of MAPK signaling and inhibition of caspase-3, -6, and -9	OE	OE, KD	UM-UC-3, EJ, T24 and RT4 cell lines	Lv et al. (2017)
hnRNP M	Splicing	Production of CD44 standard isoform, associated with poor outcome and metastases	OE	OE	Breast cancer	Sun H. et al. (2017)
**SRSFs**						
SRSF1 (SF2/ASF)	Splicing, mRNA export, mRNA NMD, nucleolar stress response, miRNA processing, mTOR activation, translation	Production of cyclin D1b isoform promoting cell proliferation	OE	KD	Prostate cancer	Olshavsky et al. (2010)
SRSF2 (SC35)	Splicing, mRNA export, transcription	Production of GCH1-L and STK39-L isoforms increasing cell growth and colony forming efficiency	OE	OE, KD	Huh7 cell lines	Luo et al. (2017)
SRSF3 (SRp20)	Splicing, translation, mRNA export and decay	Inhibition of apoptosis by down-regulating PDCD4	OE	KD	SW480 and U2OS cell lines	Kim et al. (2014)
SRSF5 (SRp40)	Splicing, translation	Induction of MCM2 and MCM4 expression, enhancing cell proliferation and colony formation efficiency	OE	KD	CAL 27 and SCC-9 cell line	Yang et al. (2018)
SRSF6 (SRp55)	Splicing	Regulation of CRH-R1 production and cell proliferation	OE	KD	Breast cancer	Lal et al. (2013)
SRSF7 (9G8)	Splicing, mRNA transport, translation	Production of exon 6 deleted Fas variant promoting cell survival	OE	KD	HCT116 and A549 cell lines	Fu and Wang (2018)
SRSF9 (SRp30c)	Splicing, RNA editing	Enhanced expression of β-catenin	OE	OE, KD	HCT116 and SW620 cell lines	Fu et al. (2013)
SRSF10 (SRp38)	Splicing	Production of BCLAF1-L variant promoting cell proliferation and growth	OE	KD	RKO and HCT116 cell lines	Zhou et al. (2014)
**Others**						
Tra2β	Splicing	Production of NASP-T isoform enhancing the HSPA2 ATPase, which increases proliferation and reduced apoptosis	OE	KD	PC-3 cell line	Alekseev et al. (2011)
Sam68	Splicing, translation, transcription, mRNA export	Production of constitutively active androgen receptor V7 variant	OE	OE, KD	LNCaP cell line	Stockley et al. (2015)

*OE, overexpression; KD, knock-down.*

The hnRNP D, that is overexpression in Eca-109 esophageal cancer cell line, has been recognized to bind the 3′-UTR AU-rich motifs of GTP cyclohydrolase (GCH1) transcripts causing GCH1 overexpression and enhanced cell proliferation and colony formation (Gao et al., 2016). Moreover, the hnRNP D protein can specifically bind to single-stranded d(TTAGGG)n human telomeric repeats through its C-terminal binding domain (BD2), thus impeding the formation of a DNA-quadruplex while favoring telomeres elongation and maintenance (Enokizono et al., 2005). The ectopic expression of p42 and/or p45 hnRNP D isoforms in hnRNP D-deleted mouse embryonic fibroblasts demonstrated that such isoforms are able to bind TERT promoter and to strongly transactivate TERT expression, thus reducing cell senescence (Pont et al., 2012).

The hnRNP E1 plays a major role in the production of integrin β1A isoform, whose expression has been found to correlate with lymph node and hepatic metastasis of pancreatic cancer, while the upregulation of hnRNP E2 was shown to promote the expression of CDK2 causing increased proliferation of gastric cancer cells (Jiang et al., 2017; Chen C. et al., 2018).

The hnRNP K expression has been demonstrated to enhance the transcription of genes involved in the extracellular matrix composition, especially MMP3 and MMP10, as well as of genes responsible for cell motility, such as PTGS2 and ITGA6, and angiogenesis, like CTGF and RASA1 (Gao R. et al., 2013).

The hnRNP L was shown to activate MAPK signaling while inhibiting caspase-3, -6 and -9 in bladder cancer cells as well as to suppress p53 expression and bcl-2/caspase-9/3 signaling via TP53 mRNA and bcl-2 binding, respectively, in prostate cancer cells (Lv et al., 2017; Zhou et al., 2017).

Overexpressed hnRNP M has been demonstrated to mediate the reduction of CD44v6 and increase of the CD44 standard isoform, that is associated with shorter overall survival and axillary lymph node metastases in breast cancer patients (Sun H. et al., 2017).

Among the SRSFs family, the SRSF1 gene was the first to be identified as a proto-oncogene with overexpression in colon, thyroid, small intestine, kidney and lung tumors (Karni et al., 2007). In particular, Karni et al. (2007) showed that SRSF1 regulates the alternative splicing of the tumor suppressor BIN1 and kinases MNK2 and S6K1 causing the production of a BIN1 isoform lacking tumor-suppressor activity, a MNK2 isoform promoting MAP kinase-independent eIF4E phosphorylation, and an oncogenic S6K1 isoform in transformed NIH 3T3 and Rat1 cell lines. The overexpression of SRSF1 was also shown to increase cell proliferation through the production of the exon 5 lacking-variant cyclin D1b in prostate cancer and to induce epithelial mesenchymal transition by stimulating the production of the ΔRon variant in breast cancer (Ghigna et al., 2005; Olshavsky et al., 2010).

Thereafter, several other SR proteins have been demonstrated to possess oncogenic activities in several tumor types (Table 1). For example, the knockdown of SRSF2 in Huh7 liver cancer cell line revealed 966 splicing alterations in cancer-related gene transcripts, favoring the production of oncogenic mRNA variants, such as the GCH1-L and STK39-L isoforms, that are involved in cell-cycle control and DNA repair (Luo et al., 2017).

The overexpression of SRSF3 into “normal” lung cell line WI-38 was able to modify the alternative splicing of the interleukin enhancer-binding factor 3 (ILF3) pre-mRNA and to increase the production of isoform-1 and isoform-2, both required for cell proliferation (Jia et al., 2019). Moreover, SRSF3 is able to deregulate the apoptotic pathway by binding to 5′-UTR of PDCD4 mRNA and by inhibiting the expression of the PDCD4, a critical suppressor of apoptosis (Kim et al., 2014).

The SRSF 5-7 proteins are particularly abundant in small cell lung cancer and associated with pleural metastasis (Kim et al., 2016). The SRSF5 enhances the expression of the MCM family members MCM2 and MCM4, that are involved in the initiation and elongation processes of DNA replication, as well as cell growth and colony formation when transfected in CAL 27 and SCC-9 oral squamous cell carcinoma cell lines (Yang et al., 2018). The SRSF6 is involved in the abnormal alternative splicing of the corticotropin-releasing hormone receptor type 1 (CRH-R1) causing indirect perturbation of oncogenic kinases, such as p38 MAPK, Akt and GSK3β, and accumulation of β-catenin in breast cancer (Lal et al., 2013). Knockdown experiments of SRSF7 in HCT116 colon and A549 lung cancer cell lines revealed the production of an exon 6-lacking isoform of the Fas receptor mRNA, that promotes cell survival (Fu and Wang, 2018).

The SRSF9 protein is frequently overexpressed in glioblastoma, colon adenocarcinoma, squamous cell lung carcinoma and malignant melanoma (Fu et al., 2013). The enhanced co-expression of SRSF9 and SRSF1 has been shown to promote tumourigenesis by Wnt signaling activation in a mTOR-dependent manner and to enhance translation of β-catenin mRNA (Fu et al., 2013).

The SRSF10 (SRp38) is an atypical SR protein acting as a potent general splicing repressor in its dephosphorylated form and as a sequence-specific splicing activator in its phosphorylated status (Feng et al., 2008). It was shown to mediate the expression of the BCLAF1 exon5a isoform (BCLAF1-L) that promotes cell proliferation and growth in colon cancer cell lines RKO and HCT116 (Zhou et al., 2014).

### Other Oncogenic Splicing Factors

Splicing factors other than hnRNPs and SR family members, such as Tra2β, Brm and Sam68, have been also demonstrated to play a role in cancer development. The Tra2β is an SR-like protein characterized by a double N- and C-terminal RS domains that is able to promote alternative exons inclusion in a dose-dependent manner (Tacke et al., 1998; Stoilov et al., 2004; Venables et al., 2005; Grellscheid et al., 2011; Elliott et al., 2012). The TRA2B gene is amplified in several tumor types, including lung, head and neck, ovary, stomach and uterus cancers (Gabriel et al., 2009; Gao J. et al., 2013). Increased levels of Tra2β promote the production of the NASP-T isoform, enhances the ATPase activity of heat shock protein HSPA2 and causes increased proliferation as well as reduced apoptosis in PC-3 prostate cancer cell line (Alekseev et al., 2011; Best et al., 2013).

The Brm is a member of SWI/SNF (mating-type switch/sucrose non-fermentable) proteins family, involved in chromatin structure remodeling and DNA-damage response (Sudarsanam and Winston, 2000; Roberts and Orkin, 2004; Smith-Roe et al., 2015). The Brm factor interacts with U1, U3, and U5 snRNPs of the major spliceosome complex and favors the inclusion of alternative exons in gene transcripts involved in carcinogenesis (Smith-Roe et al., 2015; Pulice and Kadoch, 2016). In particular, the overexpression of BRM gene enhances the inclusion of exon 9 in the E-caderin mRNA in the MCF-7 human breast cancer cell line, while the Brm knockdown promotes the production of a cyclin D1b variant lacking exon 27 in Caco2 cell lines (Batsche et al., 2006).

The Sam68 is the prototypic member of the STAR family proteins that regulates alternative splicing and RNA processing in response to signaling pathways (Taylor et al., 1995). This factor contains a 200 amino acids long domain (GRP33/SAM68/GLD-1, GSG) that bind to RNA sequences as well as C-terminal six-proline-rich and tyrosine-rich sequences that interact with SRC homology 2 (SH2) and 3 (SH3) domains of BRK, FYN and Itk/Tec/BTK kinases (Andreotti et al., 1997; Derry et al., 2000; Paronetto et al., 2003; Najib et al., 2005). Sam68 was found overexpressed and associated with poor prognosis in several cancer types, including non-small cell lung carcinoma and breast, hepatocellular, renal, prostate and gastric cancers (Busa et al., 2007; Zhang et al., 2009, 2014, 2015; Song et al., 2010; Xiao et al., 2018). In addition, it was shown to modulate the splicing of transcripts encoded by genes involved in oncogenic pathways (Bielli et al., 2011). In particular, Paronetto et al. (2010) demonstrated that ERK-phosphorylated Sam68 was able to inhibit the U1 snRNP recruitment to the intron-exon 4 junction of CCND1 mRNA in the presence of the rs9344 (870A>G) polymorphism, thus favoring the production of the cyclin D1b. Moreover, Sam68 was also demonstrated to bind an ESE located near the cryptic exon 3b 3′ splice site of the androgen receptor mRNA, promoting the inclusion of exon 3b in the transcript and its translation into the androgen receptor V7 variant in the LNCaP prostate cancer cell line (Stockley et al., 2015).

## Alternative Splicing in HPV-Related Cervical Neoplasia

The cell splicing factors contribute to cervical neoplasia by two distinct but converging mechanisms: (1) by mediating the differential maturation of HPV RNA isoforms, required for virus replication and for viral oncoprotein expression; and (2) by promoting the production of cell mRNA variants and proteins with oncogenic functions that may have roles in cervical neoplasia development and in the progression to later stages of cervical carcinogenesis (Mole et al., 2009a; Johansson and Schwartz, 2013; Oltean and Bates, 2014) (Table 2).

**TABLE 2:** Splicing factors that have been recognized to affect splicing of HPV16 and host cell transcripts in cervical cancer.

Splicing factors (aliases)	Cancer hallmarks	References	HPV16 mRNAs	References
**hnRNPs**				
hnRNP A1	Regulation of apoptosis by procaspase-3 and PARP cleavage	Patry et al. (2003)	Production of E6*I/E7 isoform, inhibition of SA5639 and of L1 mRNAs production	Cheunim et al. (2008); Rosenberger et al. (2010)
hnRNP A2/B1	Up-regulation of p21 and p27, enhanced cleavage of caspase-3, down-regulation of p-Akt	Shi et al. (2018)	Production of E6*I/E7 isoform, inhibition of SA5639 and of L1 mRNAs production	Rosenberger et al. (2010); Li et al. (2013b)
hnRNP C	–	–	Activation of SD3632 and of L1 mRNAs production	Dhanjal et al. (2015)
hnRNP D	–	–	Inhibition of SD3632 and of late mRNAs production	Li et al. (2013b)
hnRNP E1/E2	–	–	Inhibition of L2 mRNAs production	Chaudhury et al. (2010)
hnRNP F	Production of ENOX2 exon 4 minus splice variant, promoting cell growth	Tang et al. (2011)	–	–
hnRNP G	–	–	Activation of SA3358 and late mRNAs production	Yu et al. (2018)
hnRNP H	–	–	Activation of pA_E_ and inhibition of late mRNAs production	Oberg et al. (2005); Zheng et al. (2013)
hnRNP I (PTB)	Regulation of cell proliferation, anchorage-independent growth and invasiveness	Wang et al. (2008)	Inhibition of pA_L_, activation of SD3632 and of late mRNAs production	Somberg et al. (2008)
hnRNP K	Regulation of cell cycle	Lu and Gao (2016)	Inhibition of L2 mRNAs production	Collier et al. (1998)
hnRNP L	–	–	Activation of SA3358 and pA_E_, inhibition of late mRNAs production	Kajitani et al. (2017)
hnRNP P2	Promotion of EMT and cell proliferation	Zhu et al. (2018)	–	–
**SRSFs**				
SRSF1 (ASF/SF2)	Up-regulation of caspase 9a/9b ratio	Shultz et al. (2011)	Activation of SA3358 and of E6/E7 mRNA production, production of E4, E5, L1, and L2 mRNAs, inhibition of SA3632 and of late mRNAs production, inhibition of SA2709 and of E2 mRNA production	Rush et al. (2005); Somberg and Schwartz (2010); Li et al. (2013a); McFarlane et al. (2015)
SRSF2 (SC35)	Reduction of apoptosis, increased anchorage-independent growth, cell cycle progression	McFarlane et al. (2015)	Production of E6/E7 mRNAs	McFarlane et al. (2015)
SRSF3 (SRp30)	Enhancement of cell proliferation	Jia et al. (2010)	Production of E6/E7 mRNA and of E4/L1 mRNA, inhibition of SA3358 and of late mRNAs production	Rush et al. (2005); McFarlane et al. (2015); Klymenko et al. (2016)
SRSF9 (SRp30c)	Increased colony formation and proliferation, reduced apoptosis	Zhang et al. (2019a)	Inhibition of SA3358, activation of SA5639 and of late mRNAs production	Somberg et al. (2011)
SRSF10 (SRp38)	Increased cell proliferation and tumor growth, production of mIL1RAP, activation of IL-1β signal transduction and NF-κB, immune evasion	Liu et al. (2018)	–	–
SRSF11 (SRp54)	Telomeres elongation	Lee et al. (2015)	–	–
**Others**				
Tra2β	Increased lymph node metastatization, tumor grade, size and invasion depth	Gabriel et al. (2009)	–	–
Brm	Production of pro-metastatic CD44v5 in cooperation with Sam68	Batsche et al. (2006)	Production of E6/E7 mRNA	Rosenberger et al. (2010)
Sam68	Increased lymph node metastases and EMT, production of CD44v5 and of anti-apoptotic DEx3 variant	Li et al. (2012)	Production of E6/E7 mRNA	Rosenberger et al. (2010)

### HPV Infection and Viral Gene Expression

Persistent infection with high-risk HPVs, most commonly HPV16 and HPV18, is the main risk factor for development of almost all cases of cervical cancer (Doorbar, 2006; Schiffman et al., 2016), and of significant fraction of penile and anal cancers (Moscicki and Palefsky, 2011), vulvar cancer (Preti et al., 2020), as well as tumors of the upper respiratory tract, including head and neck cancers (Bzhalava et al., 2020). Most HPV infections of the cervix are successfully cleared by the host immune system in 2–3 years, but in several cases the infection may persist over the time leading to CIN graded 1 to 3 that eventually evolve during a long lasting period in invasive cervical carcinoma (McCredie et al., 2008; Doorbar et al., 2012; Oyervides-Munoz et al., 2018).

**FIGURE 4:**
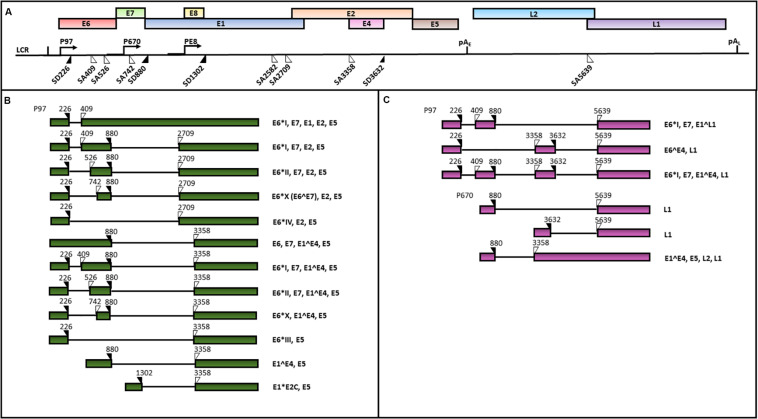
A schematic representation of the HPV16 genome and transcripts. The scheme is based on the HPV episteme (van et al., 2017). **(A)** The P97 derived early mRNAs are polyadenylated at the early polyadenylation site (pAE). The P670 and PE8 derived late mRNAs are polyadenylated at the late polyadenylation site (pAL). Both the early **(B)** and late **(C)** transcripts are polycistronic and subjected to alternative splicing (Zheng and Baker, 2006; Graham, 2010; Graham and Faizo, 2017). The donor (DS) and the acceptor (AS) splice sites in the HPV16 genome are indicated as black and white triangles, respectively. The coding potential of each transcript is also indicated.

HPV genomes are double stranded circular DNAs of approximately 8000 bp which can be divided into three main regions: (1) the early region, containing the E1, E2, E3, E4, E5, E6 and E7 ORFs, encoding for regulatory proteins; (2) the late region, including L1 and L2 ORFs, encoding for viral capsid proteins and 3) the LCR, comprising the origin of replication and multiple transcription factor binding sites (Zheng and Baker, 2006; Doorbar et al., 2015). An early polyadenylation site (pA_E_) is located between the early and the late ORFs, while a late polyadenylation site (pA_L_) is positioned between the late genes and the LCR sequence (Zheng and Baker, 2006; Graham, 2010) (Figure 4).

The HPV 16 genome contains three promoters: 1) the early promoter P97 located in the LCR region driving the transcription of early polycistronic RNAs; 2) the late promoter P670 within the E7 ORF driving the expression of late polycistronic transcripts and 3) the recent discovered E8 promoter within the ORF E8, regulating the production of the E8^E2 transcript which has been demonstrated to control the viral copy number in undifferentiated keratinocytes (Schmitt and Pawlita, 2011; Chen et al., 2014; Straub et al., 2015) (Figure 4).

The HPV gene expression during the viral life cycle is dependent on cellular differentiation stages (Doorbar, 2005). Briefly, the E1 and E2 proteins as well as the E6 and E7 proteins are expressed at low levels in the basal cell stratum of the cervical epithelium and become overexpressed once the keratinocytes move from the basal to the mid layers (Doorbar, 2006; Xue et al., 2010; Doorbar et al., 2012). The L1 and L2 genes, encoding viral capsid proteins, are only produced in the terminally differentiated layers of the cervical epithelium (Doorbar et al., 1997, 2012). In high grade CIN and cervical cancer the viral genome usually integrates into host genome, causing disruption of the E2 ORF, E6 and E7 up-regulation as well as loss of late proteins expression (Collins et al., 2009; Oyervides-Munoz et al., 2018).

### Splicing of HPV16 Transcripts

The balanced production of HPV16 early and late proteins is necessary for completion of the viral life cycle. The viral polycistronic transcripts are processed by cell splicing factors in order to remove introns through the differential usage of alternative splice sites and the production of viral proteins isoforms (Graham and Faizo, 2017). In particular, splicing sites SD226, SD880, SA409, SA526, SA742, SA2582, SA2709, and SA3358 in viral pre-mRNAs generated from the P97 promoter are recognized by the major spliceosome complex of the epithelial cells (Johansson and Schwartz, 2013). The SA3358 is the most used 3′ splice site, since these spliced mRNAs can either be polyadenylated at pA_E_ to generate mRNAs encoding E6, E7, E4, or E5, or polyadenylated at pA_L_ to produce mRNAs encoding L2 structural protein (Schmitt et al., 2010; Wu et al., 2017) (Figures 4B,C).

At late stages of productive viral infection, the binding of splicing factors to SD880, SD3632 and SA5639 sites in the transcripts generated from the P670 regulates late splicing events to produce E1^E4^L1 mRNAs, encoding both E4 and L1 proteins as well as the E1^E4 mRNAs (Johansson and Schwartz, 2013).

Alternative splicing plays a major role in the regulation of the HPV16 E6 and E7 oncogene expression. In fact, the E6 and E7 ORFs are transcribed as E6/E7 bicistronic mRNAs that can be spliced into diverse isoforms, such as E6^∗^I, E6^∗^II, E6^∗^III, E6^∗^IV, E6^∗^V, E6^∗^VI, E6^E7 (E6^∗^X), E6^E7^∗^I, and E6^E7^∗^II (Ajiro and Zheng, 2015; Brant et al., 2018; Olmedo-Nieva et al., 2018). Among these, the E6^∗^I and E6^∗^II, resulting from the SD226^SA409 and SD226^SA526 splicing, respectively, are the two major E6 isoforms expressed in cervical cancer (Zheng and Baker, 2006; Ajiro et al., 2012; Cerasuolo et al., 2017) (Figure 4B). McFarlane et al. (2015) analyzed the E6/E7 mRNA splicing pattern in cell subclones with transformed or differentiated phenotype obtained from the W12 cell line, originally derived from a HPV16-positive low grade cervical lesion (Doorbar et al., 1990). The analysis revealed the concomitant presence of E6, E6^∗^I and E6^∗^II in both transformed and differentiated W12 cell clones. However, the E6^∗^X isoform was only detected in W12 transformed cells, suggesting that the E6/E7 RNA splicing pattern may depend on specific cell transformation stages (McFarlane et al., 2015).

The alternative splicing of viral transcripts is also important to maximize the coding potential of the HPV16 genome. In fact, while the unspliced E6/E7 mRNA encodes for the full length E6 protein, the E6^∗^I and E6^∗^II mRNAs may favor the E7 expression by a termination-reinitiation process or leaky scanning mechanisms and the production of shorter E6 peptides (Zheng et al., 2004; Tang et al., 2006; Filippova et al., 2014). The E6 and E7 oncoproteins play a major role in cervical carcinogenesis for their ability to abrogate the functions of p53 and pRb oncosuppressors, respectively (Moody and Laimins, 2010; Tornesello et al., 2018; Yeo-Teh et al., 2018). Moreover, recent studies showed that the HPV16 E6^∗^I isoform have oncogenic activities, such as the disruption of mitochondrial functions and promotion of ROS production (Williams et al., 2014; Evans et al., 2016). Such activities are abrogated by the full-length E6 protein suggesting that the HPV-related cell transformation is regulated by the concerted expression of diverse E6 isoforms (Paget-Bailly et al., 2019). Further studies are needed to understand the precise role of viral mRNA splicing processes, especially E6/E7 mRNA, in high grade cervical neoplasia and cervical carcinoma.

### The Role of Splicing Factors in HPV RNAs Splicing

The splicing of HPV RNAs is modulated by cellular hnRNPs and SRSFs factors, that recognize specific enhancer and silencers motifs in the viral transcripts (Graham and Faizo, 2017; Wu et al., 2017) (Table 3).

**TABLE 3:** Binding motifs recognized by hnRNPs and SRSFs on HPV16 transcripts.

Splicing factors*	Binding motifs (5′–3′)	HPV binding regions	References
**hnRNPs**			
hnRNP A1	CAGGGU	L1	Zhao et al. (2004); Zhao and Schwartz (2008)
hnRNP A2/B1	AUAGUA	E4	Li et al. (2013b)
hnRNP C	Poly-U	Early 3′-UTR	Dhanjal et al. (2015)
hnRNP D	AUAGUA	E4	Li et al. (2013b)
hnRNP E1/E2	Poly-C	L2	Collier et al. (1998)
hnRNP G	CCGAAGAA	E4	Yu et al. (2018)
hnRNP H	GGG-repeats	L2	Oberg et al. (2005)
hnRNP I (PTB)	Poly-U	Early 3′-UTR	Zhao et al. (2005)
hnRNP K	Poly-C	L2	Collier et al. (1998)
hnRNP L	CA-repeats	E4, L1	Kajitani et al. (2017)
**SRSFs**			
SRSF1	ACCGAAGAA	E4	Somberg and Schwartz (2010)
SRSF3	ACACC, CCACACCAC	E4	Jia et al. (2009)
SRSF9	CCGAAGAA	E4	Somberg et al. (2011)

*Splicing factors whose exact binding site has not been determined yet were not reported in the table.

**FIGURE 5:**
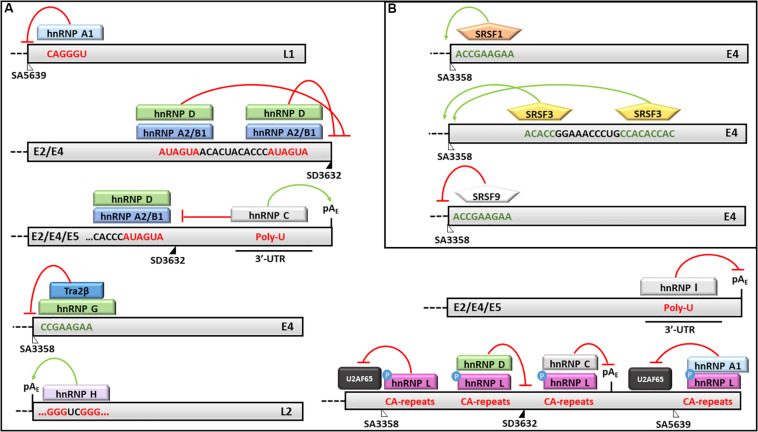
Splicing regulation of HPV16 mRNAs. Activities of **(A)** hnRNPs and **(B)** SRSFs and their binding motifs on HPV16 transcripts are shown. In particular: red lines indicate silencing activities; green arrows indicate enhancing activities; red sequences are silencing elements; while green sequences are enhancer elements. The donor (SD) and acceptor (AS) splice sites are indicated as black and white triangles, respectively.

The hnRNP A1 was shown to favor the production of the E6^∗^I isoform in the absence of EGFR, although its binding site on the E6/E7 transcript has not yet been identified (Rosenberger et al., 2010). On the other hand, the hnRNP A1 was shown to bind an ESS in the L1 coding region and to inhibit the production of L1 protein (Zhao et al., 2004, 2007; Cheunim et al., 2008; Zhao and Schwartz, 2008) (Figure 5). Recently, Ajiro et al. (2016b) demonstrated that hnRNP A1 regulates the splicing of HPV18 transcripts by binding to the ESS located at nucleotides 612-639 thus inhibiting the removal of the intron comprised between SD233 and SA416 in the E6/E7 mRNA.

Similarly, the hnRNP A2/B1 has been shown to mediate the production of the E6^∗^I isoform and to inhibit the splicing at SA5639 by preventing the binding of the U2AF65 splicing factor (Rosenberger et al., 2010; Kajitani et al., 2017). Moreover, the hnRNP A2/B1 and hnRNP D factors were demonstrated to bind two AUAGUA motifs located upstream the SD3632 and to inhibit L1 mRNA maturation and protein production in HeLa and C33A2 cervical cancer cell lines transfected with HPV16 complete genome (Li et al., 2013b) (Figure 5).

On the other hand, the hnRNP C1 has been demonstrated to increase the L1 and L2 mRNA levels through its binding to the HPV16 early untranslated region and to the AUAGUA silencer upstream the SD3632 in HPV16 -transfected HeLa and C33A cells (Dhanjal et al., 2015) (Figure 5). Recently, Nilsson et al. (2018) showed that the alkylating cancer drug melphalan induces the inhibition of HPV16 transcripts early poly-adenylation, by inducing hnRNP C interaction with the pA_E_ site, and the expression of HPV16 late gene by inhibiting CPSF30-mediated suppression of the pA_L_.

Furthermore, the hnRNP E1/E2 were shown to interact with hnRNP K and to block the L2 mRNA translation by binding to a *cis*-acting inhibitory sequence located at the 3′-end of the L2 transcript (Collier et al., 1998; Chaudhury et al., 2010).

The hnRNP G has recently been shown to bind a 8-nucleotide sequence (CCGAAGAA) located downstream the SA3358 in HPV16, thus promoting the production of late mRNAs and skipping of the exon comprised between SA3358 and SD3632 in the L1 mRNA (Yu et al., 2018) (Figure 5).

The hnRNP H was shown to promote the polyadenylation at pA_E_ site and to prevent late genes transcription in HeLa cells transfected with sub-genomic HPV16 plasmids through its interaction with the cut stimulating factor (CStF-64) and with G-rich motifs located in the L2 ORF (Oberg et al., 2005) (Figure 5). The late protein L1 was shown to interact with hnRNP H and to activate late genes transcription in differentiated keratinocytes (Zheng et al., 2013).

The hnRNP I binds a U-rich region in the 3′-UTR of HPV16 genome and induces L1/L2 mRNAs production by interfering with a polyadenylation signal at the pA_E_ and with splicing inhibitory elements located upstream and downstream the SD3632 (Zhao et al., 2005; Somberg et al., 2008) (Figure 5).

Kajitani et al. (2017) showed that the hnRNP L phosphorylation by Akt causes its association with HPV16 late splice sites (SA3358, SD3632) and pA_E_, the inhibition of U2AF65 binding and decreased L1 mRNA production. Accordingly, the siRNA mediated knockdown of hnRNP L restored the expression of HPV16 L1 and L2 proteins confirming the this splicing factor performs a fine tuning of HPV16 late transcripts (Kajitani et al., 2017) (Figure 5).

The SRSF1 factor has been shown to bind the 8-nucleotide purine-rich enhancer downstream the SA3358 site in the E4 ORF and to activate the splicing of early transcripts (except E1 and E2) as well as late mRNAs in different phases of the viral life cycle (Rush et al., 2005; Milligan et al., 2007; Somberg and Schwartz, 2010; Li et al., 2013a) (Figure 5). In addition, the SRSF1 is able to bind the exon between SA3358 and SD3632 sites causing partial inhibition of the splicing at SA3632 and production of L1 mRNAs (Somberg and Schwartz, 2010). The SRSF1 has also been shown to reduce the splicing at SA2709 causing inhibition of E2 mRNA production while favoring production of E6 and E7 transcripts in the early phases of HPV16 life cycle (Somberg and Schwartz, 2010).

The SRSF2 and SRSF3 factors promote the early viral transcripts maturation, while their knockdown induces a strong reduction of E6 mRNA isoforms and E6 and E7 oncoproteins expression in CaSki cell lines (McFarlane et al., 2015; Klymenko et al., 2016). Interestingly, the transcriptional activity of the P97 promoter was not affected by SRSF2 levels suggesting it may favor the accumulation of E6/E7 RNAs by regulating the nonsense mediated decay (McFarlane et al., 2015). In addition, SRSF3 depletion causes reduction of E4/L1 mRNA and L1 protein production in W12E cells (McFarlane et al., 2015; Klymenko et al., 2016). Moreover, the SRSF3 is able to bind an A/C-rich enhancer downstream the SA3358 and to promote early genes expression and polyadenylation while inhibiting the late genes expression in CaSki and HeLa cells (Rush et al., 2005; Jia et al., 2009) (Figure 5).

The SRSF9 was also shown to inhibit the HPV16 SA3358 enhancer and to promote the production of L1 mRNAs through the skipping of the exon between SA3358 and SD3632 sites as well as through the activation of the splicing at SA5639 (Somberg et al., 2011) (Figure 5).

Finally, the cooperation of Sam68 and Brm has been shown to promote the production of full-length E6/E7 transcripts following the activation of Sam68 by Erk1/2 kinase in the presence of EGF in E6-transfected immortalized foreskin keratinocytes (Rosenberger et al., 2010).

### HPV Proteins and Cell Splicing Factors

The HPV E2 protein has been shown to possess SRSF-like activities and to regulate viral transcription and replication thus playing a key role in the HPV life cycle (Hegde, 2002; Graham, 2016). An interactome analysis showed that E2 protein binds to several RNA processing proteins and spliceosome components, such as EFTUD2, EIF4A3, SRPK1 and SRPK2, suggesting that it may be involved in the mRNA maturation processes (Jang et al., 2015). Bodaghi et al. (2009) showed that E2 binds to SA408 in the E6/E7 pre-mRNA and interacts with SRSF5, SRSF4, SRSF6 and SRSF9 through its C-terminal domain causing intron 1 exclusion from E6 and E7 mRNAs. Moreover, in mid layers HPV infected keratinocytes the levels of E2 correlate with the expression of SR proteins and both of them increase in cervical neoplasia during CIN progression from grade 1 to grade 3 (Coupe et al., 2012).

On the other hand, chromatin immunoprecipitation assays showed that E2 binds a region comprised between nucleotides 565-363 upstream the SRSF1 AUG starting site through its N-terminal domain and transactivates the SRSF1 expression in the U2OS human osteosarcoma cell line (Mole et al., 2009b). The E2 protein has also been shown to highly transactivate the SRSF3 promoter in the E2-gene transfected U2OS cells (Klymenko et al., 2016). Mole et al. (2020) recently observed that E2-transfected keratinocytes showed an increased expression of SRPK1 and hyper-phosphorylation of SRSF1, that translocate from the nucleus to the cytoplasm in a keratinocyte differentiation-specific manner.

Furthermore, the HPV16 E6 oncoprotein was demonstrated to interact with SRSF4, SRSF6, SRSF9 and with the E6/E7 pre-mRNA promoting intron 1 retention through a NLS3 sequence localized within its C-terminal domain (Bodaghi et al., 2009).

In HPV infected cells the release of E2F1 transcription factor, caused by the E7 binding to pRb, and its recruitment to binding sites in the SRSF10 promoter region determined increased expression of SRSF10 (Liu et al., 2018). Accordingly, the silencing of E6/E7 mRNA was shown to cause downregulation of SRSF10 in cervical cancer as well as in cervical cancer cell line SiHa (Liu et al., 2018).

### Splicing Factors Deregulation in Cervical Cancer

The hnRNPs and SRSFs are generally upregulated in the basal and middle layers of the cervical epithelium and are downregulated in the terminal differentiated layers, while several of them have been found hyper-expressed in cervical carcinoma and suggested to play important roles in cervical carcinogenesis (Fay et al., 2009; Johansson and Schwartz, 2013).

In particular, the hnRNP A1 is significantly overexpressed either in high grade CIN or in cervical carcinoma compared to normal cervical epithelium representing a candidate diagnostic biomarker of cervical cancer (Kim et al., 2017; Qing et al., 2017). Interestingly, a transcriptome analysis of HPV16 E6 and E7 immortalized epithelial cells showed up-regulation of hnRNP A1 in cells cultured under hypoxic condition, suggesting that its expression could be induced by stress (Denko et al., 2000). The hnRNP A1 facilitates the production of mature miR-18a thus promoting proliferation and invasion of SiHa cells (Dong P. et al., 2019). The miR-18a suppresses the expression of PTEN, WNK2, SOX6, BTG3, and RBSP3 genes by binding to their 3′-UTR transcripts, and causes PD-L1 expression and Wnt/β-catenin pathway activation in cervical carcinoma (Dong P. et al., 2019). Moreover, the siRNA-mediated hnRNP A1/A2 knockdown caused shortening of telomeres, procaspase-3 and PARP cleavage as well as increased apoptosis in HeLa cells, suggesting that it plays a significant role in cervical cancer cell viability (Patry et al., 2003).

The role of hnRNP A2/B1 in cervical cancer has been recently investigated (Li et al., 2014). Interestingly, Li et al. (2014) observed that lobaplatin induced cell cycle arrest and apoptosis via downregulation of hnRNP A2/B1 in Caski cells. Moreover, the hnRNP A2/B1 knockdown was shown to suppress cell proliferation, migration and invasion, while increasing the apoptosis and the sensitivity to irinotecan or lobaplatin through the up-regulation of p21, p27 and cleaved caspase-3 as well as p-AKT down-regulation in HeLa and CaSki cell lines (Shi et al., 2018).

The hnRNP E1 and E2 expression decreases during the progress from low grade to high grade cervical neoplasia with an inverse correlation with HPV16 E6 protein levels (Pillai et al., 2003). Indeed, both factors may act as oncosuppressors, being under-expressed in CIN 2-3 and cervical carcinoma compared to CIN 1 (Gao et al., 2020).

Tang et al. (2011) demonstrated that hnRNP F binds to a GGGA ESS motif, located within ENOX2 exon 4, causing enhanced production of ENOX2 exon 4 minus splicing variant, that is overexpressed in cervical cancer and involved in increased cell growth.

The levels of hnRNP H and I are higher in cervical cancer and high grade CIN compared to low grade CIN and normal tissues (Oberg et al., 2005; Fay et al., 2009). The hnRNP I knockdown reduces the proliferation and anchorage-independent growth while increasing invasiveness of HeLa cells (Wang et al., 2008). The hnRNP I was also shown to regulate gene expression by forming a dimer with PTB-associated splicing factor (PSF) in SiHa and CaSki cells (Zhang et al., 2020). Moreover, Zhang et al. (2020) demonstrated that lncRNA ARAP1-AS1 interacts with PSF causing the release of hnRNP I, which in turns enhances the c-Myc IRES-dependent translation by binding to c-Myc mRNA 5′-UTR enhancing SiHa and Caski cell proliferation.

The hnRNP P2 (FUS/TLS) is highly expressed in cervical cancer as well as in SiHa and HeLa cells, dependently from the activation of XIST lncRNA/miR-200a axis, and induces the epithelial mesenchymal transition phenotype and proliferation while inhibiting apoptosis in cervical cancer cells (Zhu et al., 2018).

The SRSF1, SRSF2 and SRSF3 proteins are overexpressed in CIN 1 upper differentiated layers, in the CIN3 entire epithelium as well as in W12 cells (Mole et al., 2009a). The SRSF1 down-regulation causes the caspase 9a/9b decreased ratio and increased sensitivity of cancer cells to DNA damaging agents (Shultz et al., 2011). Similarly, the SRSF2 depletion causes increased p53 expression and apoptosis in W12 transformed subclones as well as decreased anchorage-independent growth and cell cycle arrest at G2/M checkpoint both in W12 and C33A cells (McFarlane et al., 2015). Wu et al. (2010) first demonstrated that SRSF1 was able to bind to CGGACAC motifs in the pri-miR-7-1, pri-miR-221 and pri-miR-222 stem loops thus enhancing Drosha cleavage and accumulation of miR-7, miR-221 and miR-222, promoting cervical cancer development (Pan et al., 2019). Moreover, Dong M. et al. (2019) demonstrated that the lncRNA MIR205HG, which is overexpressed in cervical cancer, targets SRSF1 and up-regulates KRT17 causing cell proliferation and apoptosis inhibition in CaSKi, Hela, MS751 and SiHa cells. The silencing of SRSF3 in HeLa cells was shown to promote the production of ILF3 isoforms 1 and 2 as well as cell cycle progression to the S and G2/M phases and proliferation enhancement (Jia et al., 2019). In addition, a miRNA array analysis showed that SRSF3 knockdown in HeLa cells caused the down-regulation of miR-16, miR-18a, miR-21, miR-92b, miR-128, miR-182, miR-629, miR-629, miR-1180, and miR-1308, and the up-regulation of miR-7, miR-26a, miR-30a, miR-99a, miR100, miR-125b, miR-181a, miR-206, miR-378, and miR-923, all involved in carcinogenic processes (Ajiro et al., 2016a; Tornesello et al., 2020).

The SRSF9 is overexpressed in cervical carcinoma, due to the down regulation of miRNA-802 that in normal epithelium binds the 3′-UTR region of SRSF9 transcript inhibiting translation, and causes increase in cell proliferation (Zhang et al., 2019b).

Similarly, the SRSF10 is increasingly overexpressed in CIN and cervical cancer cells. Its expression is higher in HPV16 and HPV18-positive compared to HPV-negative cervical cancers, but becomes strongly downregulated following the E6/E7 silencing in CaSki cells (Liu et al., 2018). Moreover, the injection of SRSF10-knockdown SiHa cells in nude mice reduced tumor growth suggesting that this factor promotes cell proliferation (Liu et al., 2018). The overexpression of SRSF10 has shown to favor the transcription of the CD47 immune evasion signal and the production of the mIL1RAP isoform, which induces the NF-κB activation and the IL-1β signal transduction associated with production of pro-inflammatory cytokines (Liu et al., 2018).

Finally, the SRSF11 (SRp54) has been observed to interact with TERC and TRF2 factors and to promote the telomerase association with telomeric repeats and telomeres elongation in HeLa cells (Lee et al., 2015).

### Other Splicing Factors in Cervical Cancer

Other proteins involved in splicing processes, including Tra2β, Brm and Sam68, have been found deregulated in cervical cancer and derived cell lines and proposed to have significant roles in cervical carcinogenesis. Specifically, the over expression of Tra2β protein, which localizes to the cervical carcinoma cell nuclei, was demonstrated to correlate with lymph node metastasis, higher tumor grade, size and depth of invasion (Gabriel et al., 2009; Best et al., 2013). The Brm factor is able to interact with ERK phosphorylated Sam68 causing the production of the CD44v5 variant by its direct binding to the exon 5 splice-regulatory elements and exon 5 retention in CD44 mRNA in HeLa cell line (Doorbar, 2005).

The Sam68 is highly expressed in cervical carcinoma and its cytoplasmic localization is shown to be associated with pelvic lymph node metastasis and poor prognosis in patients with early-stage cervical cancer (Li et al., 2012). Li et al. (2012) also demonstrated that down-regulation of Sam68 in cervical cancer cells reduced the cell motility and invasion as well as reversed the epithelial mesenchymal transition phenotype through the inhibition of the Akt/GSK-3β/Snail pathway. In HeLa cells the Sam68 was shown to bind a UAAAAAGCAU sequence within the exon 3 of survivin mRNA thus promoting the production of the anti-apoptotic DEx3 variant. Such variant has been found over-expressed in cervical adenocarcinoma HeLa cell line as well as in cervical carcinoma tissues (Futakuchi et al., 2007; Gaytan-Cervantes et al., 2017). Moreover, Sam68 was observed to interact with SRm160, a SR-rich splicing co-activator, to stimulate the inclusion of the v5 exon into the CD44 mRNA in a Ras-signaling dependent manner causing increased invasiveness of HeLa cells (Cheng and Sharp, 2006).

### Splicing-Targeted Therapeutic Strategies

The relevant role of aberrant splicing in carcinogenesis highlights the need for novel splicing-targeted therapies. Currently, different strategies have been adopted to target deregulated splicing factors and abnormal splicing variants, including the usage of small bacteria-derived molecules against the core spliceosome, splicing regulators inhibitors and anti-sense oligonucleotides against oncogenic mRNA isoforms (Lee and Abdel-Wahab, 2016; Bates et al., 2017; Lin, 2017; Di et al., 2019; Bonnal et al., 2020) (Table 4).

**TABLE 4:** Splicing-targeted strategies.

Splicing-targeted strategies	Molecules	Targets	Activity	References
Bacteria-derived compounds	Spliceostatins	SF3b	Inhibition of spliceosome assembly, splicing disruption and cell cycle arrest	Kaida et al. (2007); Zhang et al. (2019a)
	Pladienolides			
Splicing regulators inhibitors	SPHINX, SRPIN340	SRPK1	Reduced phosphorylation, altered localization and activity of SRSFs	Lu et al. (2014); Araki et al. (2015); Mavrou et al. (2015); Chang et al. (2017); Otsuka et al. (2018)
	Cpd-1, Cpd-2, Cpd-3	Clk1, Clk2		
	Resveratrol, caffeine, theophylline	hnRNPs and SRSFs mRNAs	Inhibition of hnRNPs and SRSFs expression, rescuing of aberrant splicing events and reduction of cell proliferation	
Splice-switching antisense oligonucleotides (SSOs)	2′-*O*-methylated SSO	Splicing factors binding sites (i.e., splicing acceptor and donor sited, splicing enhancers and silencers)	Inhibition of splicing factors interaction with binding sites by steric hindrance and reduction of oncogenic mRNA isoforms production	Bauman et al. (2010); Mogilevsky et al. (2018)
	2′-*O*-methoxyethylated SSO			

Several bacterial derived small molecules or their synthetic analogs have been used to inhibit the spliceosome assembly or the post-translational modifications of the core spliceosome components (Effenberger et al., 2017). Particularly, spliceostatins and pladienolides derived from *Pseudomonas* and *Streptomyces*, respectively, have shown to bind the SF3b component of U2 snRNP causing the inhibition of the spliceosome assembly and cell cycle arrest at G1 and G2/M phases (Nakajima et al., 1996; Sakai et al., 2002, 2004). The binding of spliceostatin A to SF3b1 has been demonstrated to stimulate the production of unspliced p27 mRNA encoding the C-terminal truncated p27^∗^ variant which causes cell cycle arrest at G1 phase by inhibiting CDK2 in HeLa cells (Kaida et al., 2007; Satoh and Kaida, 2016). Treatment with pladienolide B caused reduction of SF3b1 expression in a dose-dependent manner, cell cycle arrest at the G2/M phase and induction of apoptosis by increasing the expression of Tap73, cytochrome C and pro-apoptotic Bax, while decreasing the levels of ΔNp73 and anti-apoptotic Bcl-2 in HeLa cells (Zhang et al., 2019a).

Targeting of kinases regulating the splicing factors activity, such as SRPK1 and CLK2, represents a further promising therapeutic model (Urbanski et al., 2018). Either SRPK1 siRNA knockdown or treatment by SPHINX and SRPIN340 inhibitors reduced the SRSF1, SRSF2 and SRSF5 phosphorylation causing increased production of the anti-apoptotic VEGF165b isoform in PC-3 prostate cancer cell line (Mavrou et al., 2015). Araki et al. (2015) showed that Cpd-1, Cpd-2, and Cpd-3 compounds inhibit the Clk1 and Clk2 kinases activity causing reduction of SRSF4 and SRSF6 phosphorylation and modifying genome-wide splicing patterns. In particular, the transcriptome analyses performed after treatment with Cpd-2 showed changes in the splicing patterns of gene transcripts involved in growth and survival, such as EGFR, CD44, EIF3H, AURKA, HDAC1, and PARP, leading to reduced proliferation and increased apoptosis in colorectal cancer COLO205 and COLO320DM, lung cancer NCI-H23 as well as breast cancer MDA-MB-468 cell lines (Araki et al., 2015).

Splice-switching antisense oligonucleotides (SSOs) are synthetic 15–30 nucleotide long sequences that bind matched splice sites and inhibit the splicing factors interaction by steric hindrance (Havens and Hastings, 2016). Chemical modifications, such as 2′-*O*-methylation and 2′-*O*-methoxyethylation are necessary to prevent degradation of pre-mRNA-SSO complexes by RNase H (Rigo et al., 2014). The 2′-*O*-methoxyethylated Bcl-x SSO oligonucleotide is able to target the 5′-splice site in exon 2 of Bcl-x mRNA causing switch from anti-apoptotic Bcl-xL to pro-apoptotic Bcl-xS in a dose dependent manner and apoptosis via PARP cleavage in B16F10 mouse melanoma cells (Bauman et al., 2010). Furthermore, 2′-*O*-methylated SSO, targeting the 3′-splice site in exon 14b of MKNK2 mRNA, enhances the tumor-suppressor Mnkn2a isoform levels, leading to increased phosphorylation and activation of p38α–MAPK and overexpression of cFOS, COX2 and IL-6 target genes (Mogilevsky et al., 2018). The treatment with 2′-*O*-methylated SSO causes reduction of anchorage independent growth and survival in glioblastoma U87MG, hepatocellular carcinoma HuH7 and breast cancer MDA–MB–231 cell lines (Mogilevsky et al., 2018).

The resveratrol has been shown to regulate the miR-424 and miR-503 expression that inhibit hnRNP A1 production and cell proliferation in human breast cancer cell line MCF7 (Otsuka et al., 2018). In addition, bortezomib has found to reduce the expression of hnRNP K, hnRNP H, Hsp90α, Grp78, and Hsp7C in Burkytt lymphoma cell lines such as CA46 and Daudi, as determined by a proteomic analysis (Suk et al., 2015).

A natural compound, caffeine, was demonstrated to reduce the SRSF3 levels by causing increased expression of the p53β variant, reduced expression of SRSF3 targets, such as HIF-1α, SREBP1c, COX-2, FASN, and EGFR, and enhanced cell senescence (Lu et al., 2014). Similarly, theophylline down-regulates SRSF3 expression and switches the production from p53α to p53β isoform in HeLa and MCF-7 cell lines causing apoptosis, senescence, and decreased colony formation (Chang et al., 2017). Another natural drug namely indacaterol was shown to rescue SRSF6 associated aberrant splicing events, such as ZO-1 exon 23 skipping, and to reduce the viability of RKO, HCT116, and HCT8 colorectal cancer cells as well as to inhibit cancer growth in a colorectal cancer mouse model (Wan et al., 2019).

Aberrant protein isoforms have also been proposed as potential therapeutic targets (Le et al., 2015). A comprehensive genomic analysis of 38’028 tumors allowed the discovery of recurrent somatic mutations in the splice donor and acceptor sites in MET transcripts causing skipping of exon 14 in lung adenocarcinoma (Frampton et al., 2015). The expression of exon 14-lacking MET in NIH3T3 cells conferred higher sensitivity to MET-specific inhibitors, such as capmatinib, compared to cells expressing wild type MET (Frampton et al., 2015).

## Conclusion

In recent years, there is a growing experimental evidence of the key role played by deregulated splicing in cancer development and progression. Several genome-wide studies have revealed tumor specific splicing profiles at mRNA and protein level, identifying a multitude of aberrant splice variants with oncogenic functions. Different protein isoforms have been demonstrated to have a prognostic value and to modify the sensitivity of cancer cells to chemotherapeutic agents. On the other hand, therapies targeting deregulated splicing factors or pathogenic splicing isoforms are under development. Several data have been published on the role of splicing factors in cervical cancer development and further studies are needed to clarify the relevance of such factors in the production of viral or cell genome encoded oncogenic isoforms as well as in the promotion of pre-neoplastic lesions progression to invasive cancer. Much more needs to be done for the development and the clinical application of splicing-targeted therapeutic agents that would inhibit cervical cancer as well as other HPV-related tumors by affecting both cell oncogenic pathways as well as HPV life cycle.

## Author Contributions

AC performed bibliography analysis and wrote the manuscript. LB and FB supervised the whole project. MT designed the study and drafted the manuscript. All authors read and approved the final manuscript.

## Conflict of Interest

The authors declare that the research was conducted in the absence of any commercial or financial relationships that could be construed as a potential conflict of interest.

## Abbreviations

BARD1: BRCA1 associated RING domain 1
BCLAF1: BCL2 associated transcription factor 1
BIN1: bridging integrator-1
BRCA1, breast cancer 1: early onset
BRM: brahma
BTG3: BTG anti-proliferation factor 3
Casp: caspase
CCAR1: cell cycle and apoptosis regulator 1
CCK: cholecystokinin
CCND1: cyclin D1
CDK: cyclin dependent kinase
CFTR: cystic fibrosis transmembrane conductance regulator
CIN: cervical intraepithelial neoplasia
Clk/Sty kinases: Cdc2-like kinase/Ser-Thr-Tyr kinase
COX-2: cyclooxygenase 2
CPSF30: cleavage polyadenylation specificity factor 30
CRH-R1: corticotropin-releasing hormone receptor type 1
CTGF: connective tissue growth factor
E2F1: E2F transcription factor 1
EFTUD2: elongation factor Tu GTP binding domain containing 2
EGFR: epidermal growth factor receptor
EIF3H: eukaryotic translation initiation factor 3 subunit H
EIF4A3: eukaryotic translation initiation factor 4A3
eIF4E: eukaryotic translation initiation factor 4E
EMT: epithelial-mesenchymal transition
ERK: extracellular signal-regulated kinase
ESE: exonic splicing enhancer
ESS: exonic splicing silencer
FASN: fatty acid synthase
FOXO4: forkhead box O4
GATA3: GATA binding protein 3
GCH1: GTP cyclohydrolase 1
GLUT1: glucose transporter
GSK3 β: glycogen synthase kinase 3 beta
HDAC1: histone deacetylase 1
HIF-1 α: hypoxia-inducible factor-1
hnRNP: heterogeneous nuclear ribonucleoprotein
HPV: human papillomavirus
Hsp90 α /7C: heat shock protein 90 α /7C
HSPA2: heat shock protein family A
IL1RAP: interleukin 1 receptor accessory protein
ILF3: interleukin enhancer-binding factor 3
ISE: intronic splicing enhancer
ISS: intronic splicing silencer
ITGA6: integrin subunit alpha 6
KH domain: K homology domain
LCR: long control region
LDHA: lactate dehydrogenase A
lncRNA: long non-coding RNA
MAPK: mitogen-activated protein kinase
MCM2/4: minichromosome maintenance complex component 2/4
miR: micro-RNA
MKNK2: MAPK interacting serine/threonine kinase 2
MMP3: matrix metallopeptidase 3
mTOR: mechanistic target of rapamycin kinase
NASP: nuclear autoantigenic sperm protein
NCL: nucleolin
NEK2: NIMA related kinase 2
NF- κ B: nuclear factor kappa-light-chain-enhancer of activated B cells
NLS: nuclear localization signal
NMD: non-sense mediated decay
ORF: open reading frame
pAE/pAL: early/late polyadenylation signal
PARP: poly ADP-ribose polymerase
PCNA: proliferating cell nuclear antigen
PDCD4: programmed cell death 4
PDK1: pyruvate dehydrogenase kinase 1
pRb: retinoblastoma protein
Pre-mRNA: pre-messenger RNA
PTEN: phosphatase and tensin homolog
PTGS2: prostaglandin-endoperoxide synthase 2
RAC1: ras-related C3 botulinum toxin substrate 1
RASA1: RAS protein activator 1
Ron: recepteur d’origine nantais
RRM: RNA recognition motif
Sam68: Src-associated in mitosis 68 kDa
S6K2: S6 kinase 2
SD/SA: splice donor/acceptor site
SH2/SH3: Sarc homology 2/3
snRNP: small nuclear ribonucleoprotein
SR protein: serine/arginine-rich protein
SREBP1c: sterol regulatory element-binding transcription factor 1
SRPK: serine/arginine kinase
SRSF: serine/arginine-rich splicing factor
STAR: signal transduction and activation of RNA
STAT: signal transducer and activator of transcription
STK: serine/threonine kinase
SUMO1: small ubiquitin-like modifiers 1
SWI/SNF: SWItch/sucrose non-fermentable
TCGA: The Cancer Genome Atlas
TERC: telomerase RNA component
TOPO1: topoisomerase 1
TRA2B: transformer-2 protein homolog beta
TRF2: telomeric repeat-binding factor 2
U2AF65: U2 small nuclear RNA auxiliary factor 2
UTR: untranslated region
VEGF: vascular endothelial growth factor
WNK2: WNK lysine deficient protein kinase 2
XIAP: X-linked inhibitor of apoptosis
XIST: X inactive specific transcript
ZO-1: tight junction protein-1.

## References

[B1] Aebi M. Hornig H. Padgett R. A. Reiser J. Weissmann C. (1986). Sequence requirements for splicing of higher eukaryotic nuclear pre-mRNA. *Cell* 47 555–565. 10.1016/0092-8674(86)90620-3 3779836

[B2] Ajiro M. Jia R. Yang Y. Zhu J. Zheng Z. M. (2016a). A genome landscape of SRSF3-regulated splicing events and gene expression in human osteosarcoma U2OS cells. *Nucleic Acids Res.* 44 1854–1870. 10.1093/nar/gkv1500 26704980 PMC4770227

[B3] Ajiro M. Jia R. Zhang L. Liu X. Zheng Z. M. (2012). Intron definition and a branch site adenosine at nt 385 control RNA splicing of HPV16 E6^∗^I and E7 expression. *PLoS One* 7:e46412. 10.1371/journal.pone.0046412 23056301 PMC3464268

[B4] Ajiro M. Tang S. Doorbar J. Zheng Z. M. (2016b). Serine/Arginine-rich splicing factor 3 and heterogeneous nuclear ribonucleoprotein A1 regulate alternative RNA splicing and gene expression of human papillomavirus 18 through two functionally distinguishable cis elements. *J. Virol.* 90 9138–9152. 10.1128/jvi.00965-16 27489271 PMC5044842

[B5] Ajiro M. Zheng Z. M. (2015). E6^E7, a novel splice isoform protein of human papillomavirus 16, stabilizes viral E6 and E7 oncoproteins via HSP90 and GRP78. *mBio* 6:e02068-14. 10.1128/mBio.02068-14 PMC4337564 25691589

[B6] Alekseev O. M. Richardson R. T. Tsuruta J. K. O’Rand M. G. (2011). Depletion of the histone chaperone tNASP inhibits proliferation and induces apoptosis in prostate cancer PC-3 cells. *Reprod. Biol. Endocrinol.* 9:50. 10.1186/1477-7827-9-50 21496299 PMC3100250

[B7] Anczukow O. Krainer A. R. (2016). Splicing-factor alterations in cancers. *RNA* 22 1285–1301. 10.1261/rna.057919.116 27530828 PMC4986885

[B8] Andreotti A. H. Bunnell S. C. Feng S. Berg L. J. Schreiber S. L. (1997). Regulatory intramolecular association in a tyrosine kinase of the Tec family. *Nature* 385 93–97. 10.1038/385093a0 8985255

[B9] Araki S. Dairiki R. Nakayama Y. Murai A. Miyashita R. Iwatani M. (2015). Inhibitors of CLK protein kinases suppress cell growth and induce apoptosis by modulating pre-mRNA splicing. *PLoS One* 10:e0116929. 10.1371/journal.pone.0116929 25581376 PMC4291223

[B10] Arbab J. P. Ayatollahi H. Sadeghi R. Sheikhi M. Asghari A. (2018). Prognostic significance of SRSF2 mutations in myelodysplastic syndromes and chronic myelomonocytic leukemia: a meta-analysis. *Hematology* 23 778–784. 10.1080/10245332.2018.1471794 29757120

[B11] Aubol B. E. Keshwani M. M. Fattet L. Adams J. A. (2018). Mobilization of a splicing factor through a nuclear kinase-kinase complex. *Biochem. J.* 475 677–690. 10.1042/bcj20170672 29335301 PMC6293969

[B12] Aujla A. Linder K. Iragavarapu C. Karass M. Liu D. (2018). SRSF2 mutations in myelodysplasia/myeloproliferative neoplasms. *Biomark. Res.* 6:29. 10.1186/s40364-018-0142-y PMC6158887 30275952

[B13] Auyeung V. C. Ulitsky I. McGeary S. E. Bartel D. P. (2013). Beyond secondary structure: primary-sequence determinants license pri-miRNA hairpins for processing. *Cell* 152 844–858. 10.1016/j.cell.2013.01.031 23415231 PMC3707628

[B14] Baralle F. E. Giudice J. (2017). Alternative splicing as a regulator of development and tissue identity. *Nat. Rev. Mol. Cell Biol.* 18 437–451. 10.1038/nrm.2017.27 28488700 PMC6839889

[B15] Bates D. O. Morris J. C. Oltean S. Donaldson L. F. (2017). Pharmacology of modulators of alternative splicing. *Pharmacol. Rev.* 69 63–79. 10.1124/pr.115.011239 28034912 PMC5226212

[B16] Batsche E. Yaniv M. Muchardt C. (2006). The human SWI/SNF subunit Brm is a regulator of alternative splicing. *Nat. Struct. Mol. Biol.* 13 22–29. 10.1038/nsmb1030 16341228

[B17] Bauman J. A. Li S. D. Yang A. Huang L. Kole R. (2010). Anti-tumor activity of splice-switching oligonucleotides. *Nucleic Acids Res.* 38 8348–8356. 10.1093/nar/gkq731 20719743 PMC3001088

[B18] Best A. Dagliesh C. Ehrmann I. Kheirollahi-Kouhestani M. Tyson-Capper A. Elliott D. J. (2013). Expression of Tra2 beta in cancer cells as a potential contributory factor to neoplasia and metastasis. *Int. J. Cell Biol.* 2013:843781. 10.1155/2013/843781 PMC3723085 23935626

[B19] Bielli P. Busa R. Paronetto M. P. Sette C. (2011). The RNA-binding protein Sam68 is a multifunctional player in human cancer. *Endocr. Relat. Cancer* 18 R91–R102. 21565971 10.1530/ERC-11-0041

[B20] Bodaghi S. Jia R. Zheng Z. M. (2009). Human papillomavirus type 16 E2 and E6 are RNA-binding proteins and inhibit in vitro splicing of pre-mRNAs with suboptimal splice sites. *Virology* 386 32–43. 10.1016/j.virol.2008.12.037 19187948 PMC2683163

[B21] Bonnal S. C. Lopez-Oreja I. Valcarcel J. (2020). Roles and mechanisms of alternative splicing in cancer – implications for care. *Nat. Rev. Clin. Oncol.* 10.1038/s41571-020-0350-x [Epub ahead of print]. 32303702

[B22] Boukakis G. Patrinou-Georgoula M. Lekarakou M. Valavanis C. Guialis A. (2010). Deregulated expression of hnRNP A/B proteins in human non-small cell lung cancer: parallel assessment of protein and mRNA levels in paired tumour/non-tumour tissues. *BMC Cancer* 10:434. 10.1186/1471-2407-10-434 20716340 PMC2933625

[B23] Bradley T. Cook M. E. Blanchette M. (2015). SR proteins control a complex network of RNA-processing events. *RNA* 21 75–92. 10.1261/rna.043893.113 25414008 PMC4274639

[B24] Brant A. C. Menezes A. N. Felix S. P. de Almeida L. M. Sammeth M. Moreira M. A. M. (2018). Characterization of HPV integration, viral gene expression and E6E7 alternative transcripts by RNA-Seq: a descriptive study in invasive cervical cancer. *Genomics* 111 1853–1861. 10.1016/j.ygeno.2018.12.008 30552977

[B25] Buljan M. Chalancon G. Eustermann S. Wagner G. P. Fuxreiter M. Bateman A. (2012). Tissue-specific splicing of disordered segments that embed binding motifs rewires protein interaction networks. *Mol. Cell* 46 871–883. 10.1016/j.molcel.2012.05.039 22749400 PMC3437557

[B26] Busa R. Paronetto M. P. Farini D. Pierantozzi E. Botti F. Angelini D. F. (2007). The RNA-binding protein Sam68 contributes to proliferation and survival of human prostate cancer cells. *Oncogene* 26 4372–4382. 10.1038/sj.onc.1210224 17237817

[B27] Busch A. Hertel K. J. (2012). Evolution of SR protein and hnRNP splicing regulatory factors. *Wiley Interdiscip. Rev. RNA* 3 1–12. 10.1002/wrna.100 21898828 PMC3235224

[B28] Bush S. J. Chen L. Tovar-Corona J. M. Urrutia A. O. (2017). Alternative splicing and the evolution of phenotypic novelty. *Philos. Trans. R. Soc. Lond. B Biol. Sci.* 2017:372. 10.1098/rstb.2015.0474 PMC5182408 27994117

[B29] Bzhalava Z. Arroyo Muhr L. S. Dillner J. (2020). Transcription of human papillomavirus oncogenes in head and neck squamous cell carcinomas. *Vaccine* 38 4066–4070. 10.1016/j.vaccine.2020.04.049 32362526

[B30] Cerasuolo A. Annunziata C. Tortora M. Starita N. Stellato G. Greggi S. (2017). Comparative analysis of HPV16 gene expression profiles in cervical and in oropharyngeal squamous cell carcinoma. *Oncotarget* 8 34070–34081. 10.18632/oncotarget.15977 28423662 PMC5470952

[B31] Chang Y. L. Hsu Y. J. Chen Y. Wang Y. W. Huang S. M. (2017). Theophylline exhibits anti-cancer activity via suppressing SRSF3 in cervical and breast cancer cell lines. *Oncotarget* 8 101461–101474. 10.18632/oncotarget.21464 29254178 PMC5731888

[B32] Chaudhury A. Chander P. Howe P. H. (2010). Heterogeneous nuclear ribonucleoproteins (hnRNPs) in cellular processes: focus on hnRNP E1’s multifunctional regulatory roles. *RNA* 16 1449–1462. 10.1261/rna.2254110 20584894 PMC2905745

[B33] Che Y. Fu L. (2020). Aberrant expression and regulatory network of splicing factor-SRSF3 in tumors. *J. Cancer* 11 3502–3511. 10.7150/jca.42645 32284746 PMC7150454

[B34] Chen C. Lei J. Zheng Q. Tan S. Ding K. Yu C. (2018). Poly(rC) binding protein 2 (PCBP2) promotes the viability of human gastric cancer cells by regulating CDK2. *FEBS Open Bio.* 8 764–773. 10.1002/2211-5463.12408 29744291 PMC5929926

[B35] Chen H. Liu H. Qing G. (2018). Targeting oncogenic Myc as a strategy for cancer treatment. *Signal. Transduct. Target Ther.* 3:5. 10.1038/s41392-018-0008-7 PMC5837124 29527331

[B36] Chen J. Xue Y. Poidinger M. Lim T. Chew S. H. Pang C. L. (2014). Mapping of HPV transcripts in four human cervical lesions using RNAseq suggests quantitative rearrangements during carcinogenic progression. *Virology* 46 14–24. 10.1016/j.virol.2014.05.026 25092457

[B37] Chen M. Manley J. L. (2009). Mechanisms of alternative splicing regulation: insights from molecular and genomics approaches. *Nat. Rev. Mol. Cell Biol.* 10 741–754. 10.1038/nrm2777 19773805 PMC2958924

[B38] Chen Y. Huang Q. Liu W. Zhu Q. Cui C. P. Xu L. (2018a). Mutually exclusive acetylation and ubiquitylation of the splicing factor SRSF5 control tumor growth. *Nat. Commun.* 9:2464. 10.1038/s41467-018-04815-3 PMC6018636 29942010

[B39] Chen Y. Liu J. Wang W. Xiang L. Wang J. Liu S. (2018b). High expression of hnRNPA1 promotes cell invasion by inducing EMT in gastric cancer. *Oncol. Rep.* 39 1693–1701. 29484423 10.3892/or.2018.6273 PMC5868405

[B40] Cheng C. Sharp P. A. (2006). Regulation of CD44 alternative splicing by SRm160 and its potential role in tumor cell invasion. *Mol. Cell Biol.* 26 362–370. 10.1128/mcb.26.1.362-370.2006 16354706 PMC1317625

[B41] Cheng Z. Sun Y. Niu X. Shang Y. Ruan J. Chen Z. (2017). Gene expression profiling reveals U1 snRNA regulates cancer gene expression. *Oncotarget* 8 112867–112874. 10.18632/oncotarget.22842 29348872 PMC5762557

[B42] Cheunim T. Zhang J. Milligan S. G. McPhillips M. G. Graham S. V. (2008). The alternative splicing factor hnRNP A1 is up-regulated during virus-infected epithelial cell differentiation and binds the human papillomavirus type 16 late regulatory element. *Virus Res.* 131 189–198. 10.1016/j.virusres.2007.09.006 17950949 PMC2635527

[B43] Christofk H. R. Vander Heiden M. G. Harris M. H. Ramanathan A. Gerszten R. E. Wei R. (2008). The M2 splice isoform of pyruvate kinase is important for cancer metabolism and tumour growth. *Nature* 452 230–233. 10.1038/nature06734 18337823

[B44] Climente-Gonzalez H. Porta-Pardo E. Godzik A. Eyras E. (2017). The functional impact of alternative splicing in cancer. *Cell Rep.* 20 2215–2226. 10.1016/j.celrep.2017.08.012 28854369

[B45] Collier B. Goobar-Larsson L. Sokolowski M. Schwartz S. (1998). Translational inhibition in vitro of human papillomavirus type 16 L2 mRNA mediated through interaction with heterogenous ribonucleoprotein K and poly(rC)-binding proteins 1 and 2. *J. Biol. Chem.* 273 22648–22656. 10.1074/jbc.273.35.22648 9712894

[B46] Collins S. I. Constandinou-Williams C. Wen K. Young L. S. Roberts S. Murray P. G. (2009). Disruption of the E2 gene is a common and early event in the natural history of cervical human papillomavirus infection: a longitudinal cohort study. *Cancer Res.* 69 3828–3832. 10.1158/0008-5472.can-08-3099 19401452

[B47] Colwill K. Pawson T. Andrews B. Prasad J. Manley J. L. Bell J. C. (1996). The Clk/Sty protein kinase phosphorylates SR splicing factors and regulates their intranuclear distribution. *EMBO J.* 15 265–275. 10.1002/j.1460-2075.1996.tb00357.x 8617202 PMC449941

[B48] Coupe V. M. Gonzalez-Barreiro L. Gutierrez-Berzal J. Melian-Boveda A. L. Lopez-Rodriguez O. Alba-Dominguez J. (2012). Transcriptional analysis of human papillomavirus type 16 in histological sections of cervical dysplasia by in situ hybridisation. *J. Clin. Pathol.* 65 164–170. 10.1136/jclinpath-2011-200330 22075186

[B49] Czubaty A. Piekielko-Witkowska A. (2017). Protein kinases that phosphorylate splicing factors: roles in cancer development, progression and possible therapeutic options. *Int. J. Biochem. Cell Biol.* 91(Pt B) 102–115. 10.1016/j.biocel.2017.05.024 28552434

[B50] Das S. Anczukow O. Akerman M. Krainer A. R. (2012). Oncogenic splicing factor SRSF1 is a critical transcriptional target of MYC. *Cell Rep.* 1 110–117. 10.1016/j.celrep.2011.12.001 22545246 PMC3334311

[B51] David C. J. Chen M. Assanah M. Canoll P. Manley J. L. (2010). HnRNP proteins controlled by c-Myc deregulate pyruvate kinase mRNA splicing in cancer. *Nature* 463 364–368. 10.1038/nature08697 20010808 PMC2950088

[B52] David J. Maden S. Weeder B. Thompson R. Nellore A. (2020). Putatively cancer-specific exonΓÇôexon junctions are shared across patients and present in developmental and other non-cancer cells. *NAR Cancer* 1:2. 10.1093/narcan/zcaa001 PMC8209686 34316681

[B53] Denko N. Schindler C. Koong A. Laderoute K. Green C. Giaccia A. (2000). Epigenetic regulation of gene expression in cervical cancer cells by the tumor microenvironment. *Clin. Cancer Res.* 6 480–487. 10690527

[B54] Derry J. J. Richard S. Valderrama C. H. Ye X. Vasioukhin V. Cochrane A. W. (2000). Sik (BRK) phosphorylates Sam68 in the nucleus and negatively regulates its RNA binding ability. *Mol. Cell Biol.* 20 6114–6126. 10.1128/mcb.20.16.6114-6126.2000 10913193 PMC86087

[B55] Dhanjal S. Kajitani N. Glahder J. Mossberg A. K. Johansson C. Schwartz S. (2015). Heterogeneous nuclear ribonucleoprotein C proteins interact with the human papillomavirus type 16 (HPV16) Early 3′-untranslated region and alleviate suppression of HPV16 late L1 mRNA splicing. *J. Biol. Chem.* 290 13354–13371. 10.1074/jbc.m115.638098 25878250 PMC4505585

[B56] Di C. Syafrizayanti Zhang Q Chen Y. Wang Y. Zhang X. (2019). Function, clinical application, and strategies of Pre-mRNA splicing in cancer. *Cell Death Differ.* 26 1181–1194. 10.1038/s41418-018-0231-3 30464224 PMC6748147

[B57] Dong M. Dong Z. Zhu X. Zhang Y. Song L. (2019). Long non-coding RNA MIR205HG regulates KRT17 and tumor processes in cervical cancer via interaction with SRSF1. *Exp. Mol. Pathol.* 111:104322. 10.1016/j.yexmp.2019.104322 31655037

[B58] Dong P. Xiong Y. Yu J. Chen L. Tao T. Yi S. (2019). Correction: control of PD-L1 expression by miR-140/142/340/383 and oncogenic activation of the OCT4-miR-18a pathway in cervical cancer. *Oncogene* 38:3972. 10.1038/s41388-019-0677-x 30679789 PMC7608127

[B59] Doorbar J. (2005). The papillomavirus life cycle. *J. Clin. Virol.* 32(Suppl. 1) S7–S15. 15753007 10.1016/j.jcv.2004.12.006

[B60] Doorbar J. (2006). Molecular biology of human papillomavirus infection and cervical cancer. *Clin. Sci.* 110 525–541. 10.1042/cs20050369 16597322

[B61] Doorbar J. Egawa N. Griffin H. Kranjec C. Murakami I. (2015). Human papillomavirus molecular biology and disease association. *Rev. Med. Virol.* 25(Suppl. 1) 2–23. 10.1002/rmv.1822 25752814 PMC5024016

[B62] Doorbar J. Foo C. Coleman N. Medcalf L. Hartley O. Prospero T. (1997). Characterization of events during the late stages of HPV16 infection in vivo using high-affinity synthetic Fabs to E4. *Virology* 238 40–52. 10.1006/viro.1997.8768 9375007

[B63] Doorbar J. Parton A. Hartley K. Banks L. Crook T. Stanley M. (1990). Detection of novel splicing patterns in a HPV16-containing keratinocyte cell line. *Virology* 178 254–262. 10.1016/0042-6822(90)90401-c 2167553

[B64] Doorbar J. Quint W. Banks L. Bravo I. G. Stoler M. Broker T. R. (2012). The biology and life-cycle of human papillomaviruses. *Vaccine* 30(Suppl. 5) F55–F70. 23199966 10.1016/j.vaccine.2012.06.083

[B65] Dvinge H. (2018). Regulation of alternative mRNA splicing: old players and new perspectives. *FEBS Lett.* 592 2987–3006. 10.1002/1873-3468.13119 29856907

[B66] Dvinge H. Kim E. Abdel-Wahab O. Bradley R. K. (2016). RNA splicing factors. *Nat. Rev. Cancer* 16 413–430. 27282250 10.1038/nrc.2016.51 PMC5094465

[B67] Effenberger K. A. Urabe V. K. Jurica M. S. (2017). Modulating splicing with small molecular inhibitors of the spliceosome. *Wiley Interdiscip. Rev. RNA* 8:10.1002/wrna.1381. 10.1002/wrna.1381 PMC5253128 27440103

[B68] El M. E. Younis I. (2018). The cancer spliceome: reprograming of alternative splicing in cancer. *Front. Mol. Biosci.* 5:80. 10.3389/fmolb.2018.00080 30246013 PMC6137424

[B69] Elliott D. J. Best A. Dalgliesh C. Ehrmann I. Grellscheid S. (2012). How does Tra2beta protein regulate tissue-specific RNA splicing? *Biochem. Soc. Trans.* 40 784–788. 10.1042/bst20120036 22817734 PMC3950818

[B70] Enokizono Y. Konishi Y. Nagata K. Ouhashi K. Uesugi S. Ishikawa F. (2005). Structure of hnRNP D complexed with single-stranded telomere DNA and unfolding of the quadruplex by heterogeneous nuclear ribonucleoprotein D. *J. Biol. Chem.* 280 18862–18870. 10.1074/jbc.m411822200 15734733

[B71] Evans W. Filippova M. Filippov V. Bashkirova S. Zhang G. Reeves M. E. (2016). Overexpression of HPV16 E6^∗^ alters beta-integrin and mitochondrial dysfunction pathways in cervical cancer cells. *Cancer Genomics Proteomics* 13 259–273. 27365376

[B72] Fay J. Kelehan P. Lambkin H. Schwartz S. (2009). Increased expression of cellular RNA-binding proteins in HPV-induced neoplasia and cervical cancer. *J. Med. Virol.* 81 897–907. 10.1002/jmv.21406 19319956

[B73] Fei T. Chen Y. Xiao T. Li W. Cato L. Zhang P. (2017). Genome-wide CRISPR screen identifies HNRNPL as a prostate cancer dependency regulating RNA splicing. *Proc. Natl. Acad. Sci. U.S.A.* 114 E5207–E5215. 28611215 10.1073/pnas.1617467114 PMC5495225

[B74] Feng Y. Chen M. Manley J. L. (2008). Phosphorylation switches the general splicing repressor SRp38 to a sequence-specific activator. *Nat. Struct. Mol. Biol.* 15 1040–1048. 10.1038/nsmb.1485 18794844 PMC2668916

[B75] Filippova M. Evans W. Aragon R. Filippov V. Williams V. M. Hong L. (2014). The small splice variant of HPV16 E6, E6, reduces tumor formation in cervical carcinoma xenografts. *Virology* 45 153–164. 10.1016/j.virol.2013.12.011 24503078 PMC3932372

[B76] Frampton G. M. Ali S. M. Rosenzweig M. Chmielecki J. Lu X. Bauer T. M. (2015). Activation of MET via diverse exon 14 splicing alterations occurs in multiple tumor types and confers clinical sensitivity to MET inhibitors. *Cancer Discov.* 5 850–859. 10.1158/2159-8290.cd-15-0285 25971938

[B77] Frendewey D. Keller W. (1985). Stepwise assembly of a pre-mRNA splicing complex requires U-snRNPs and specific intron sequences. *Cell* 42 355–367. 10.1016/s0092-8674(85)80131-8 3160483

[B78] Friend L. R. Landsberg M. J. Nouwens A. S. Wei Y. Rothnagel J. A. Smith R. (2013). Arginine methylation of hnRNP A2 does not directly govern its subcellular localization. *PLoS One* 8:e75669. 10.1371/journal.pone.0075669 24098712 PMC3787039

[B79] Fu Y. Huang B. Shi Z. Han J. Wang Y. Huangfu J. (2013). SRSF1 and SRSF9 RNA binding proteins promote Wnt signalling-mediated tumorigenesis by enhancing beta-catenin biosynthesis. *EMBO Mol. Med.* 5 737–750. 10.1002/emmm.201202218 23592547 PMC3662316

[B80] Fu Y. Wang Y. (2018). SRSF7 knockdown promotes apoptosis of colon and lung cancer cells. *Oncol. Lett.* 15 5545–5552. 29556298 10.3892/ol.2018.8072 PMC5844074

[B81] Futakuchi H. Ueda M. Kanda K. Fujino K. Yamaguchi H. Noda S. (2007). Transcriptional expression of survivin and its splice variants in cervical carcinomas. *Int. J. Gynecol. Cancer* 17 1092–1098. 10.1111/j.1525-1438.2007.00833.x 17877643

[B82] Gabriel B. Zur H. A. Bouda J. Boudova L. Koprivova M. Hirschfeld M. (2009). Significance of nuclear hTra2-beta1 expression in cervical cancer. *Acta Obstet. Gynecol. Scand.* 88 216–221. 10.1080/00016340802503021 19037821

[B83] Gallardo M. Lee H. J. Zhang X. Bueso-Ramos C. Pageon L. R. McArthur M. (2015). hnRNP K is a haploinsufficient tumor suppressor that regulates proliferation and differentiation programs in hematologic malignancies. *Cancer Cell* 28 486–499. 10.1016/j.ccell.2015.09.001 26412324 PMC4652598

[B84] Gao J. Aksoy B. A. Dogrusoz U. Dresdner G. Gross B. Sumer S. O. (2013). Integrative analysis of complex cancer genomics and clinical profiles using the cBioPortal. *Sci. Signal.* 6:pl1. 10.1126/scisignal.2004088 23550210 PMC4160307

[B85] Gao R. Yu Y. Inoue A. Widodo N. Kaul S. C. Wadhwa R. (2013). Heterogeneous nuclear ribonucleoprotein K (hnRNP-K) promotes tumor metastasis by induction of genes involved in extracellular matrix, cell movement, and angiogenesis. *J. Biol. Chem.* 288 15046–15056. 10.1074/jbc.m113.466136 23564449 PMC3663525

[B86] Gao W. Ding L. Song Z. C. Feng M. J. Liu C. L. Li X. X. (2020). [The role of human papillomavirus 16 early genes E2 and E6 and heterogeneous nuclear ribonucleoprotein E2 in cervical carcinogenesis and their interaction effect]. *Zhonghua Yu Fang Yi Xue Za Zhi* 54 92–98. 31914575 10.3760/cma.j.issn.0253-9624.2020.01.017

[B87] Gao Y. Wang W. Cao J. Wang F. Geng Y. Cao J. (2016). Upregulation of AUF1 is involved in the proliferation of esophageal squamous cell carcinoma through GCH1. *Int. J. Oncol.* 49 2001–2010. 10.3892/ijo.2016.3713 27826622

[B88] Gaytan-Cervantes J. Gonzalez-Torres C. Maldonado V. Zampedri C. Ceballos-Cancino G. Melendez-Zajgla J. (2017). Protein Sam68 regulates the alternative splicing of survivin DEx3. *J. Biol. Chem.* 292 13745–13757. 10.1074/jbc.m117.800318 28655776 PMC5566528

[B89] Geuens T. Bouhy D. Timmerman V. (2016). The hnRNP family: insights into their role in health and disease. *Hum. Genet.* 135 851–867. 10.1007/s00439-016-1683-5 27215579 PMC4947485

[B90] Ghigna C. Giordano S. Shen H. Benvenuto F. Castiglioni F. Comoglio P. M. (2005). Cell motility is controlled by SF2/ASF through alternative splicing of the Ron protooncogene. *Mol. Cell* 20 881–890. 10.1016/j.molcel.2005.10.026 16364913

[B91] Giannakouros T. Nikolakaki E. Mylonis I. Georgatsou E. (2011). Serine-arginine protein kinases: a small protein kinase family with a large cellular presence. *FEBS J.* 278 570–586. 10.1111/j.1742-4658.2010.07987.x 21205200

[B92] Golan-Gerstl R. Cohen M. Shilo A. Suh S. S. Bakacs A. Coppola L. (2011). Splicing factor hnRNP A2/B1 regulates tumor suppressor gene splicing and is an oncogenic driver in glioblastoma. *Cancer Res.* 71 4464–4472. 10.1158/0008-5472.can-10-4410 21586613

[B93] Goncalves V. Henriques A. F. Pereira J. F. Neves C. A. Moyer M. P. Moita L. F. (2014). Phosphorylation of SRSF1 by SRPK1 regulates alternative splicing of tumor-related Rac1b in colorectal cells. *RNA* 20 474–482. 10.1261/rna.041376.113 24550521 PMC3964909

[B94] Grabowski P. J. Seiler S. R. Sharp P. A. (1985). A multicomponent. *Cell* 42 345–353. 3160482 10.1016/s0092-8674(85)80130-6

[B95] Graham S. V. (2010). Human papillomavirus: gene expression, regulation and prospects for novel diagnostic methods and antiviral therapies. *Future Microbiol.* 5 1493–1506. 10.2217/fmb.10.107 21073310 PMC3527891

[B96] Graham S. V. (2016). Human papillomavirus E2 protein: linking replication, transcription, and RNA processing. *J. Virol.* 90 8384–8388. 10.1128/jvi.00502-16 27412596 PMC5021420

[B97] Graham S. V. Faizo A. A. A. (2017). Control of human papillomavirus gene expression by alternative splicing. *Virus Res.* 231 83–95. 10.1016/j.virusres.2016.11.016 27867028 PMC5335905

[B98] Grellscheid S. Dalgliesh C. Storbeck M. Best A. Liu Y. Jakubik M. (2011). Identification of evolutionarily conserved exons as regulated targets for the splicing activator tra2beta in development. *PLoS Genet.* 7:e1002390. 10.1371/journal.pgen.1002390 22194695 PMC3240583

[B99] Guil S. Caceres J. F. (2007). The multifunctional RNA-binding protein hnRNP A1 is required for processing of miR-18a. *Nat. Struct. Mol. Biol.* 14 591–596. 10.1038/nsmb1250 17558416

[B100] Habelhah H. Shah K. Huang L. Ostareck-Lederer A. Burlingame A. L. Shokat K. M. (2001). ERK phosphorylation drives cytoplasmic accumulation of hnRNP-K and inhibition of mRNA translation. *Nat. Cell Biol.* 3 325–330. 10.1038/35060131 11231586

[B101] Han S. P. Tang Y. H. Smith R. (2010). Functional diversity of the hnRNPs: past, present and perspectives. *Biochem. J.* 430 379–392. 10.1042/bj20100396 20795951

[B102] Havens M. A. Hastings M. L. (2016). Splice-switching antisense oligonucleotides as therapeutic drugs. *Nucleic Acids Res.* 44 6549–6563. 10.1093/nar/gkw533 27288447 PMC5001604

[B103] Hegde R. S. (2002). The papillomavirus E2 proteins: structure, function, and biology. *Annu. Rev. Biophys. Biomol. Struct.* 31 343–360. 10.1146/annurev.biophys.31.100901.142129 11988474

[B104] Howard J. M. Sanford J. R. (2015). The RNAissance family: SR proteins as multifaceted regulators of gene expression. *Wiley Interdiscip. Rev. RNA* 6 93–110. 10.1002/wrna.1260 25155147 PMC4268343

[B105] Jang M. K. Anderson D. E. van D. K. McBride A. A. (2015). A proteomic approach to discover and compare interacting partners of papillomavirus E2 proteins from diverse phylogenetic groups. *Proteomics* 15 2038–2050. 10.1002/pmic.201400613 25758368 PMC4535189

[B106] Jeong S. (2017). SR proteins: binders, regulators, and connectors of RNA. *Mol. Cells* 40 1–9. 10.14348/molcells.2017.2319 28152302 PMC5303883

[B107] Jia R. Ajiro M. Yu L. McCoy P. Jr. Zheng Z. M. (2019). Oncogenic splicing factor SRSF3 regulates ILF3 alternative splicing to promote cancer cell proliferation and transformation. *RNA* 25 630–644. 10.1261/rna.068619.118 30796096 PMC6467003

[B108] Jia R. Li C. McCoy J. P. Deng C. X. Zheng Z. M. (2010). SRp20 is a proto-oncogene critical for cell proliferation and tumor induction and maintenance. *Int. J. Biol. Sci.* 6 806–826. 10.7150/ijbs.6.806 21179588 PMC3005347

[B109] Jia R. Liu X. Tao M. Kruhlak M. Guo M. Meyers C. (2009). Control of the papillomavirus early-to-late switch by differentially expressed SRp20. *J. Virol.* 83 167–180. 10.1128/jvi.01719-08 18945760 PMC2612334

[B110] Jiang P. Li Z. Tian F. Li X. Yang J. (2017). Fyn/heterogeneous nuclear ribonucleoprotein E1 signaling regulates pancreatic cancer metastasis by affecting the alternative splicing of integrin beta1. *Int. J. Oncol.* 51 169–183. 10.3892/ijo.2017.4018 28560430 PMC5467783

[B111] Johansson C. Schwartz S. (2013). Regulation of human papillomavirus gene expression by splicing and polyadenylation. *Nat. Rev. Microbiol.* 11 239–251. 10.1038/nrmicro2984 23474685

[B112] Kahles A. Lehmann K. V. Toussaint N. C. Huser M. Stark S. G. Sachsenberg T. (2018). Comprehensive analysis of alternative splicing across tumors from 8,705 patients. *Cancer Cell* 34 211–224. 30078747 10.1016/j.ccell.2018.07.001 PMC9844097

[B113] Kaida D. Motoyoshi H. Tashiro E. Nojima T. Hagiwara M. Ishigami K. (2007). Spliceostatin A targets SF3b and inhibits both splicing and nuclear retention of pre-mRNA. *Nat. Chem. Biol.* 3 576–583. 10.1038/nchembio.2007.18 17643111

[B114] Kajitani N. Glahder J. Wu C. Yu H. Nilsson K. Schwartz S. (2017). hnRNP L controls HPV16 RNA polyadenylation and splicing in an Akt kinase-dependent manner. *Nucleic Acids Res.* 45 9654–9678. 10.1093/nar/gkx606 28934469 PMC5766200

[B115] Kandoth C. McLellan M. D. Vandin F. Ye K. Niu B. Lu C. (2013). Mutational landscape and significance across 12 major cancer types. *Nature* 502 333–339. 10.1038/nature12634 24132290 PMC3927368

[B116] Kanopka A. Muhlemann O. Akusjarvi G. (1996). Inhibition by SR proteins of splicing of a regulated adenovirus pre-mRNA. *Nature* 381 535–538. 10.1038/381535a0 8632829

[B117] Karni R. de S. E. Lowe S. W. Sinha R. Mu D. Krainer A. R. (2007). The gene encoding the splicing factor SF2/ASF is a proto-oncogene. *Nat. Struct. Mol. Biol.* 14 185–193. 10.1038/nsmb1209 17310252 PMC4595851

[B118] Kastner B. Will C. L. Stark H. Luhrmann R. (2019). Structural insights into nuclear pre-mRNA splicing in higher eukaryotes. *Cold Spring Harb. Perspect. Biol.* 11:a032417. 10.1101/cshperspect.a032417 30765414 PMC6824238

[B119] Kim H. R. Lee G. O. Choi K. H. Kim D. K. Ryu J. S. Hwang K. E. (2016). SRSF5: a novel marker for small-cell lung cancer and pleural metastatic cancer. *Lung Cancer* 99 57–65. 10.1016/j.lungcan.2016.05.018 27565915

[B120] Kim J. Park R. Y. Chen J. K. Kim J. Jeong S. Ohn T. (2014). Splicing factor SRSF3 represses the translation of programmed cell death 4 mRNA by associating with the 5′-UTR region. *Cell Death Differ.* 21 481–490. 10.1038/cdd.2013.171 24292556 PMC3921596

[B121] Kim Y. J. Kim B. R. Ryu J. S. Lee G. O. Kim H. R. Choi K. H. (2017). HNRNPA1, a splicing regulator, is an effective target protein for cervical cancer detection: comparison with conventional tumor markers. *Int. J. Gynecol. Cancer* 27 326–331. 10.1097/igc.0000000000000868 27984373

[B122] Klymenko T. Hernandez-Lopez H. MacDonald A. I. Bodily J. M. Graham S. V. (2016). Human papillomavirus E2 regulates SRSF3 (SRp20) to promote capsid protein expression in infected differentiated keratinocytes. *J. Virol.* 90 5047–5058. 10.1128/jvi.03073-15 26962216 PMC4859725

[B123] Kohler J. Schuler M. Gauler T. C. Nopel-Dunnebacke S. Ahrens M. Hoffmann A. C. (2016). Circulating U2 small nuclear RNA fragments as a diagnostic and prognostic biomarker in lung cancer patients. *J. Cancer Res. Clin. Oncol.* 142 795–805. 10.1007/s00432-015-2095-y 26687686 PMC11819072

[B124] Komatsu S. Ichikawa D. Takeshita H. Morimura R. Hirajima S. Tsujiura M. (2014). Circulating miR-18a: a sensitive cancer screening biomarker in human cancer. *In Vivo* 28 293–297. 24815829

[B125] Komeno Y. Huang Y. J. Qiu J. Lin L. Xu Y. Zhou Y. (2015). SRSF2 is essential for hematopoiesis, and its myelodysplastic syndrome-related mutations dysregulate alternative pre-mRNA splicing. *Mol. Cell Biol.* 35 3071–3082. 10.1128/mcb.00202-15 26124281 PMC4525309

[B126] Kooshapur H. Choudhury N. R. Simon B. Muhlbauer M. Jussupow A. Fernandez N. (2018). Structural basis for terminal loop recognition and stimulation of pri-miRNA-18a processing by hnRNP A1. *Nat. Commun.* 9:2479. 10.1038/s41467-018-04871-9 PMC6018666 29946118

[B127] Kramer M. C. Liang D. Tatomer D. C. Gold B. March Z. M. Cherry S. (2015). Combinatorial control of *Drosophila* circular RNA expression by intronic repeats, hnRNPs, and SR proteins. *Genes Dev.* 29 2168–2182. 10.1101/gad.270421.115 26450910 PMC4617980

[B128] Lai M. C. Lin R. I. Huang S. Y. Tsai C. W. Tarn W. Y. (2000). A human importin-beta family protein, transportin-SR2, interacts with the phosphorylated RS domain of SR proteins. *J. Biol. Chem.* 275 7950–7957. 10.1074/jbc.275.11.7950 10713112

[B129] Lai M. C. Lin R. I. Tarn W. Y. (2001). Transportin-SR2 mediates nuclear import of phosphorylated SR proteins. *Proc. Natl. Acad. Sci. U.S.A.* 98 10154–10159. 10.1073/pnas.181354098 11517331 PMC56931

[B130] Lal S. Allan A. Markovic D. Walker R. Macartney J. Europe-Finner N. (2013). Estrogen alters the splicing of type 1 corticotropin-releasing hormone receptor in breast cancer cells. *Sci. Signal.* 6:ra53. 10.1126/scisignal.2003926 23821771

[B131] Le K. Q. Prabhakar B. S. Hong W. J. Li L. C. (2015). Alternative splicing as a biomarker and potential target for drug discovery. *Acta Pharmacol. Sin.* 36 1212–1218. 10.1038/aps.2015.43 26073330 PMC4648177

[B132] Lee J. H. Jeong S. A. Khadka P. Hong J. Chung I. K. (2015). Involvement of SRSF11 in cell cycle-specific recruitment of telomerase to telomeres at nuclear speckles. *Nucleic Acids Res.* 43 8435–8451. 10.1093/nar/gkv844 26286192 PMC4787792

[B133] Lee S. C. Abdel-Wahab O. (2016). Therapeutic targeting of splicing in cancer. *Nat. Med.* 22 976–986. 10.1038/nm.4165 27603132 PMC5644489

[B134] Lee S. D. Yu D. Lee D. Y. Shin H. S. Jo J. H. Lee Y. C. (2019). Upregulated microRNA-193a-3p is responsible for cisplatin resistance in CD44(+) gastric cancer cells. *Cancer Sci.* 110 662–673. 10.1111/cas.13894 30485589 PMC6361556

[B135] Lee S. W. Lee M. H. Park J. H. Kang S. H. Yoo H. M. Ka S. H. (2012). SUMOylation of hnRNP-K is required for p53-mediated cell-cycle arrest in response to DNA damage. *EMBO J.* 31 4441–4452. 10.1038/emboj.2012.293 23092970 PMC3512394

[B136] Lee Y. Rio D. C. (2015). Mechanisms and regulation of alternative Pre-mRNA splicing. *Annu. Rev. Biochem.* 84 291–323. 25784052 10.1146/annurev-biochem-060614-034316 PMC4526142

[B137] Li K. Sun D. Gou Q. Ke X. Gong Y. Zuo Y. (2018). Long non-coding RNA linc00460 promotes epithelial-mesenchymal transition and cell migration in lung cancer cells. *Cancer Lett.* 420 80–90. 10.1016/j.canlet.2018.01.060 29409808

[B138] Li X. Johansson C. Cardoso P. C. Mossberg A. Dhanjal S. Bergvall M. (2013a). Eight nucleotide substitutions inhibit splicing to HPV-16 3′-splice site SA3358 and reduce the efficiency by which HPV-16 increases the life span of primary human keratinocytes. *PLoS One* 8:e72776. 10.1371/journal.pone.0072776 24039800 PMC3767658

[B139] Li X. Johansson C. Glahder J. Mossberg A. K. Schwartz S. (2013b). Suppression of HPV-16 late L1 5′-splice site SD3632 by binding of hnRNP D proteins and hnRNP A2/B1 to upstream AUAGUA RNA motifs. *Nucleic Acids Res.* 41 10488–10508. 10.1093/nar/gkt803 24013563 PMC3905901

[B140] Li X. Ran L. Fang W. Wang D. (2014). Lobaplatin arrests cell cycle progression, induces apoptosis and alters the proteome in human cervical cancer cell Line CaSki. *Biomed. Pharmacother.* 68 291–297. 10.1016/j.biopha.2013.10.004 24239273

[B141] Li Z. Yu C. P. Zhong Y. Liu T. J. Huang Q. D. Zhao X. H. (2012). Sam68 expression and cytoplasmic localization is correlated with lymph node metastasis as well as prognosis in patients with early-stage cervical cancer. *Ann. Oncol.* 23 638–646. 10.1093/annonc/mdr290 21700735

[B142] Liang Y. Tebaldi T. Rejeski K. Joshi P. Stefani G. Taylor A. (2018). SRSF2 mutations drive oncogenesis by activating a global program of aberrant alternative splicing in hematopoietic cells. *Leukemia* 32 2659–2671. 10.1038/s41375-018-0152-7 29858584 PMC6274620

[B143] Lin J. C. (2017). Therapeutic applications of targeted alternative splicing to cancer treatment. *Int. J. Mol. Sci.* 19:75. 10.3390/ijms19010075 29283381 PMC5796025

[B144] Liu F. Dai M. Xu Q. Zhu X. Zhou Y. Jiang S. (2018). SRSF10-mediated IL1RAP alternative splicing regulates cervical cancer oncogenesis via mIL1RAP-NF-kappaB-CD47 axis. *Oncogene* 37 2394–2409. 10.1038/s41388-017-0119-6 29429992 PMC5931977

[B145] Liu X. Zhou Y. Lou Y. Zhong H. (2016). Knockdown of HNRNPA1 inhibits lung adenocarcinoma cell proliferation through cell cycle arrest at G0/G1 phase. *Gene* 576(2 Pt 2) 791–797. 10.1016/j.gene.2015.11.009 26581508

[B146] Long Y. Sou W. H. Yung K. W. Y. Liu H. Wan S. W. C. Li Q. (2019). Distinct mechanisms govern the phosphorylation of different SR protein splicing factors. *J. Biol. Chem.* 294 1312–1327. 10.1074/jbc.ra118.003392 30478176 PMC6349114

[B147] Lu G. Y. Huang S. M. Liu S. T. Liu P. Y. Chou W. Y. Lin W. S. (2014). Caffeine induces tumor cytotoxicity via the regulation of alternative splicing in subsets of cancer-associated genes. *Int. J. Biochem. Cell Biol.* 47 83–92. 10.1016/j.biocel.2013.12.004 24333670

[B148] Lu J. Gao F. H. (2016). Role and molecular mechanism of heterogeneous nuclear ribonucleoprotein K in tumor development and progression. *Biomed. Rep.* 4 657–663. 10.3892/br.2016.642 27284403 PMC4887935

[B149] Luo C. Cheng Y. Liu Y. Chen L. Liu L. Wei N. (2017). SRSF2 Regulates alternative splicing to drive hepatocellular carcinoma development. *Cancer Res.* 77 1168–1178. 10.1158/0008-5472.can-16-1919 28082404

[B150] Lv D. Wu H. Xing R. Shu F. Lei B. Lei C. (2017). HnRNP-L mediates bladder cancer progression by inhibiting apoptotic signaling and enhancing MAPK signaling pathways. *Oncotarget* 8 13586–13599. 10.18632/oncotarget.14600 28088793 PMC5355122

[B151] Manley J. L. Krainer A. R. (2010). A rational nomenclature for serine/arginine-rich protein splicing factors (SR proteins). *Genes Dev.* 24 1073–1074. 10.1101/gad.1934910 20516191 PMC2878644

[B152] Masaki S. Ikeda S. Hata A. Shiozawa Y. Kon A. Ogawa S. (2019). Myelodysplastic syndrome-associated SRSF2 mutations cause splicing changes by altering binding motif sequences. *Front. Genet.* 10:338. 10.3389/fgene.2019.00338 31040863 PMC6476956

[B153] Mavrou A. Brakspear K. Hamdollah-Zadeh M. Damodaran G. Babaei-Jadidi R. Oxley J. (2015). Serine-arginine protein kinase 1 (SRPK1) inhibition as a potential novel targeted therapeutic strategy in prostate cancer. *Oncogene* 34 4311–4319. 10.1038/onc.2014.360 25381816 PMC4351909

[B154] McCredie M. R. Sharples K. J. Paul C. Baranyai J. Medley G. Jones R. W. (2008). Natural history of cervical neoplasia and risk of invasive cancer in women with cervical intraepithelial neoplasia 3: a retrospective cohort study. *Lancet Oncol.* 9 425–434. 10.1016/s1470-2045(08)70103-7 18407790

[B155] McFarlane M. MacDonald A. I. Stevenson A. Graham S. V. (2015). Human papillomavirus 16 oncoprotein expression is controlled by the cellular splicing factor SRSF2 (SC35). *J. Virol.* 89 5276–5287. 10.1128/jvi.03434-14 25717103 PMC4442513

[B156] Mili S. Shu H. J. Zhao Y. Pinol-Roma S. (2001). Distinct RNP complexes of shuttling hnRNP proteins with pre-mRNA and mRNA: candidate intermediates in formation and export of mRNA. *Mol. Cell Biol.* 21 7307–7319. 10.1128/mcb.21.21.7307-7319.2001 11585913 PMC99905

[B157] Milligan S. G. Veerapraditsin T. Ahamet B. Mole S. Graham S. V. (2007). Analysis of novel human papillomavirus type 16 late mRNAs in differentiated W12 cervical epithelial cells. *Virology* 360 172–181. 10.1016/j.virol.2006.10.012 17098271 PMC2151308

[B158] Mogilevsky M. Shimshon O. Kumar S. Mogilevsky A. Keshet E. Yavin E. (2018). Modulation of MKNK2 alternative splicing by splice-switching oligonucleotides as a novel approach for glioblastoma treatment. *Nucleic Acids Res.* 46 11396–11404. 10.1093/nar/gky921 30329087 PMC6265459

[B159] Mole S. Faizo A. A. A. Hernandez-Lopez H. Griffiths M. Stevenson A. Roberts S. (2020). Human papillomavirus type 16 infection activates the host serine arginine protein kinase 1 (SRPK1) – splicing factor axis. *J. Gen. Virol.* 10.1099/jgv.0.001402 [Epub ahead of print]. 32182205 PMC7414453

[B160] Mole S. McFarlane M. Chuen-Im T. Milligan S. G. Millan D. Graham S. V. (2009a). RNA splicing factors regulated by HPV16 during cervical tumour progression. *J. Pathol.* 219 383–391. 10.1002/path.2608 19718710 PMC2779514

[B161] Mole S. Milligan S. G. Graham S. V. (2009b). Human papillomavirus type 16 E2 protein transcriptionally activates the promoter of a key cellular splicing factor, SF2/ASF. *J. Virol.* 83 357–367. 10.1128/jvi.01414-08 18945764 PMC2612322

[B162] Moody C. A. Laimins L. A. (2010). Human papillomavirus oncoproteins: pathways to transformation. *Nat. Rev. Cancer* 10 550–560. 10.1038/nrc2886 20592731

[B163] Moscicki A. B. Palefsky J. M. (2011). Human papillomavirus in men: an update. *J. Low. Genit. Tract. Dis.* 15 231–234. 10.1097/lgt.0b013e318203ae61 21543996 PMC3304470

[B164] Moujalled D. James J. L. Yang S. Zhang K. Duncan C. Moujalled D. M. (2015). Phosphorylation of hnRNP K by cyclin-dependent kinase 2 controls cytosolic accumulation of TDP-43. *Hum. Mol. Genet.* 24 1655–1669. 10.1093/hmg/ddu578 25410660

[B165] Najib S. Martin-Romero C. Gonzalez-Yanes C. Sanchez-Margalet V. (2005). Role of Sam68 as an adaptor protein in signal transduction. *Cell. Mol. Life Sci.* 62 36–43. 10.1007/s00018-004-4309-3 15619005 PMC11924462

[B166] Nakajima H. Sato B. Fujita T. Takase S. Terano H. Okuhara M. (1996). New antitumor substances, FR901463, FR901464 and FR901465. I. Taxonomy, fermentation, isolation, physico-chemical properties and biological activities. *J. Antibiot.* 49 1196–1203. 10.7164/antibiotics.49.1196 9031664

[B167] Nakielny S. Dreyfuss G. (1996). The hnRNP C proteins contain a nuclear retention sequence that can override nuclear export signals. *J. Cell Biol.* 134 1365–1373. 10.1083/jcb.134.6.1365 8830767 PMC2121000

[B168] Naro C. Barbagallo F. Chieffi P. Bourgeois C. F. Paronetto M. P. Sette C. (2014). The centrosomal kinase NEK2 is a novel splicing factor kinase involved in cell survival. *Nucleic Acids Res.* 42 3218–3227. 10.1093/nar/gkt1307 24369428 PMC3950702

[B169] Naro C. Sette C. (2013). Phosphorylation-mediated regulation of alternative splicing in cancer. *Int. J. Cell Biol.* 2013:151839. 10.1155/2013/151839 PMC3771450 24069033

[B170] Nasim F. U. Hutchison S. Cordeau M. Chabot B. (2002). High-affinity hnRNP A1 binding sites and duplex-forming inverted repeats have similar effects on 5′ splice site selection in support of a common looping out and repression mechanism. *RNA* 8 1078–1089. 10.1017/s1355838202024056 12212851 PMC1370318

[B171] Nilsson K. Wu C. Kajitani N. Yu H. Tsimtsirakis E. Gong L. (2018). The DNA damage response activates HPV16 late gene expression at the level of RNA processing. *Nucleic Acids Res.* 46 5029–5049. 10.1093/nar/gky227 29596642 PMC6007495

[B172] Oberg D. Fay J. Lambkin H. Schwartz S. (2005). A downstream polyadenylation element in human papillomavirus type 16 L2 encodes multiple GGG motifs and interacts with hnRNP H. *J. Virol.* 79 9254–9269. 10.1128/jvi.79.14.9254-9269.2005 15994820 PMC1168734

[B173] Olmedo-Nieva L. Munoz-Bello J. O. Contreras-Paredes A. Lizano M. (2018). The role of E6 spliced isoforms (E6^∗^) in human papillomavirus-induced carcinogenesis. *Viruses* 10:45. 10.3390/v10010045 29346309 PMC5795458

[B174] Olshavsky N. A. Comstock C. E. Schiewer M. J. Augello M. A. Hyslop T. Sette C. (2010). Identification of ASF/SF2 as a critical, allele-specific effector of the cyclin D1b oncogene. *Cancer Res.* 70 3975–3984. 10.1158/0008-5472.can-09-3468 20460515 PMC2873684

[B175] Oltean S. Bates D. O. (2014). Hallmarks of alternative splicing in cancer. *Oncogene* 33 5311–5318. 10.1038/onc.2013.533 24336324

[B176] Otsuka K. Yamamoto Y. Ochiya T. (2018). Regulatory role of resveratrol, a microRNA-controlling compound, in HNRNPA1 expression, which is associated with poor prognosis in breast cancer. *Oncotarget* 9 24718–24730. 10.18632/oncotarget.25339 29872500 PMC5973863

[B177] Oyervides-Munoz M. A. Perez-Maya A. A. Rodriguez-Gutierrez H. F. Gomez-Macias G. S. Fajardo-Ramirez O. R. Trevino V. (2018). Understanding the HPV integration and its progression to cervical cancer. *Infect. Genet. Evol.* 61 134–144. 10.1016/j.meegid.2018.03.003 29518579

[B178] Pagani F. Buratti E. Stuani C. Romano M. Zuccato E. Niksic M. (2000). Splicing factors induce cystic fibrosis transmembrane regulator exon 9 skipping through a nonevolutionary conserved intronic element. *J. Biol. Chem.* 275 21041–21047. 10.1074/jbc.m910165199 10766763

[B179] Paget-Bailly P. Meznad K. Bruyere D. Perrard J. Herfs M. Jung A. C. (2019). Comparative RNA sequencing reveals that HPV16 E6 abrogates the effect of E6^∗^I on ROS metabolism. *Sci. Rep.* 9:5938. 10.1038/s41598-019-42393-6 PMC6459911 30976051

[B180] Pan Z. X. Zhang X. Y. Chen S. R. Li C. Z. (2019). Upregulated exosomal miR-221/222 promotes cervical cancer via repressing methyl-CpG-binding domain protein 2. *Eur. Rev. Med. Pharmacol. Sci.* 23 3645–3653. 31114989 10.26355/eurrev_201905_17788

[B181] Papasaikas P. Valcarcel J. (2016). The spliceosome: the ultimate RNA chaperone and sculptor. *Trends Biochem. Sci.* 41 33–45. 10.1016/j.tibs.2015.11.003 26682498

[B182] Park Y. M. Hwang S. J. Masuda K. Choi K. M. Jeong M. R. Nam D. H. (2012). Heterogeneous nuclear ribonucleoprotein C1/C2 controls the metastatic potential of glioblastoma by regulating PDCD4. *Mol. Cell Biol.* 32 4237–4244. 10.1128/mcb.00443-12 22907752 PMC3457347

[B183] Paronetto M. P. Cappellari M. Busa R. Pedrotti S. Vitali R. Comstock C. (2010). Alternative splicing of the cyclin D1 proto-oncogene is regulated by the RNA-binding protein Sam68. *Cancer Res.* 70 229–239. 10.1158/0008-5472.can-09-2788 20028857 PMC2884274

[B184] Paronetto M. P. Venables J. P. Elliott D. J. Geremia R. Rossi P. Sette C. (2003). Tr-kit promotes the formation of a multimolecular complex composed by Fyn. PLCgamma1 and Sam68. *Oncogene* 22 8707–8715. 10.1038/sj.onc.1207016 14647465

[B185] Parssinen J. Kuukasjarvi T. Karhu R. Kallioniemi A. (2007). High-level amplification at 17q23 leads to coordinated overexpression of multiple adjacent genes in breast cancer. *Br. J. Cancer* 96 1258–1264. 10.1038/sj.bjc.6603692 17353917 PMC2360139

[B186] Patel M. Sachidanandan M. Adnan M. (2019). Serine arginine protein kinase 1 (SRPK1): a moonlighting protein with theranostic ability in cancer prevention. *Mol. Biol. Rep.* 46 1487–1497. 10.1007/s11033-018-4545-5 30535769

[B187] Patry C. Bouchard L. Labrecque P. Gendron D. Lemieux B. Toutant J. (2003). Small interfering RNA-mediated reduction in heterogeneous nuclear ribonucleoparticule A1/A2 proteins induces apoptosis in human cancer cells but not in normal mortal cell lines. *Cancer Res.* 63 7679–7688. 14633690

[B188] Phoomak C. Park D. Silsirivanit A. Sawanyawisuth K. Vaeteewoottacharn K. Detarya M. (2019). O-GlcNAc-induced nuclear translocation of hnRNP-K is associated with progression and metastasis of cholangiocarcinoma. *Mol. Oncol.* 13 338–357. 10.1002/1878-0261.12406 30444036 PMC6360360

[B189] Pillai M. R. Chacko P. Kesari L. A. Jayaprakash P. G. Jayaram H. N. Antony A. C. (2003). Expression of folate receptors and heterogeneous nuclear ribonucleoprotein E1 in women with human papillomavirus mediated transformation of cervical tissue to cancer. *J. Clin. Pathol.* 56 569–574. 10.1136/jcp.56.8.569 12890803 PMC1770025

[B190] Pont A. R. Sadri N. Hsiao S. J. Smith S. Schneider R. J. (2012). mRNA decay factor AUF1 maintains normal aging, telomere maintenance, and suppression of senescence by activation of telomerase transcription. *Mol. Cell* 47 5–15. 10.1016/j.molcel.2012.04.019 22633954 PMC3966316

[B191] Preti M. Rotondo J. C. Holzinger D. Micheletti L. Gallio N. McKay-Chopin S. (2020). Role of human papillomavirus infection in the etiology of vulvar cancer in Italian women. *Infect. Agent Cancer* 15:20. 10.1186/s13027-020-00286-8 PMC7110671 32266002

[B192] Pulice J. L. Kadoch C. (2016). Composition and function of mammalian SWI/SNF chromatin remodeling complexes in human disease. *Cold Spring Harb. Symp. Quant. Biol.* 81 53–60. 10.1101/sqb.2016.81.031021 28408647

[B193] Qing S. Tulake W. Ru M. Li X. Yuemaier R. Lidifu D. (2017). Proteomic identification of potential biomarkers for cervical squamous cell carcinoma and human papillomavirus infection. *Tumour Biol.* 39 1010428317697547. 10.1177/1010428317697547 28443473

[B194] Ratnadiwakara M. Mohenska M. Anko M. L. (2018). Splicing factors as regulators of miRNA biogenesis - links to human disease. *Semin. Cell Dev. Biol.* 79 113–122. 10.1016/j.semcdb.2017.10.008 29042235

[B195] Rigo F. Seth P. P. Bennett C. F. (2014). Antisense oligonucleotide-based therapies for diseases caused by pre-mRNA processing defects. *Adv. Exp. Med. Biol.* 825 303–352. 10.1007/978-1-4939-1221-6_9 25201110

[B196] Roberts C. W. Orkin S. H. (2004). The SWI/SNF complex–chromatin and cancer. *Nat. Rev. Cancer* 4 133–142. 14964309 10.1038/nrc1273

[B197] Rodriguez-Aguayo C. Monroig P. D. C. Redis R. S. Bayraktar E. Almeida M. I. Ivan C. (2017). Regulation of hnRNPA1 by microRNAs controls the miR-18a-K-RAS axis in chemotherapy-resistant ovarian cancer. *Cell Discov.* 3:17029. 10.1038/celldisc.2017.29 PMC5594916 28904816

[B198] Rosenberger S. De-Castro A. J. Langbein L. Steenbergen R. D. Rosl F. (2010). Alternative splicing of human papillomavirus type-16 E6/E6^∗^ early mRNA is coupled to EGF signaling via Erk1/2 activation. *Proc. Natl. Acad. Sci. U.S.A.* 107 7006–7011. 10.1073/pnas.1002620107 20351270 PMC2872467

[B199] Rossi F. Labourier E. Forne T. Divita G. Derancourt J. Riou J. F. (1996). Specific phosphorylation of SR proteins by mammalian DNA topoisomerase I. *Nature* 381 80–82. 10.1038/381080a0 8609994

[B200] Rush M. Zhao X. Schwartz S. (2005). A splicing enhancer in the E4 coding region of human papillomavirus type 16 is required for early mRNA splicing and polyadenylation as well as inhibition of premature late gene expression. *J. Virol.* 79 12002–12015. 10.1128/jvi.79.18.12002-12015.2005 16140776 PMC1212645

[B201] Sakai T. Asai N. Okuda A. Kawamura N. Mizui Y. (2004). Pladienolides, new substances from culture of Streptomyces platensis Mer-11107. II. Physico-chemical properties and structure elucidation. *J. Antibiot.* 57 180–187. 10.7164/antibiotics.57.180 15152803

[B202] Sakai Y. Yoshida T. Ochiai K. Uosaki Y. Saitoh Y. Tanaka F. (2002). GEX1 compounds, novel antitumor antibiotics related to herboxidiene, produced by *Streptomyces* sp. I. Taxonomy, production, isolation, physicochemical properties and biological activities. *J. Antibiot.* 55 855–862. 10.7164/antibiotics.55.855 12523818

[B203] Satoh T. Kaida D. (2016). Upregulation of p27 cyclin-dependent kinase inhibitor and a C-terminus truncated form of p27 contributes to G1 phase arrest. *Sci. Rep.* 6:27829. 10.1038/srep27829 PMC4901259 27282251

[B204] Schiffman M. Doorbar J. Wentzensen N. de S. S. Fakhry C. Monk B. J. (2016). Carcinogenic human papillomavirus infection. *Nat. Rev. Dis. Primers* 2:16086. 10.1038/nrdp.2016.86 27905473

[B205] Schmitt M. Dalstein V. Waterboer T. Clavel C. Gissmann L. Pawlita M. (2010). Diagnosing cervical cancer and high-grade precursors by HPV16 transcription patterns. *Cancer Res.* 70 249–256. 10.1158/0008-5472.can-09-2514 20028865

[B206] Schmitt M. Pawlita M. (2011). The HPV transcriptome in HPV16 positive cell lines. *Mol. Cell Probes* 25 108–113. 10.1016/j.mcp.2011.03.003 21439369

[B207] Seiler M. Peng S. Agrawal A. A. Palacino J. Teng T. Zhu P. (2018). Somatic mutational landscape of splicing factor genes and their functional consequences across 33 cancer types. *Cell Rep.* 23 282–296. 29617667 10.1016/j.celrep.2018.01.088 PMC5933844

[B208] Shen M. Mattox W. (2012). Activation and repression functions of an SR splicing regulator depend on exonic versus intronic-binding position. *Nucleic Acids Res.* 40 428–437. 10.1093/nar/gkr713 21914724 PMC3245930

[B209] Shi X. Ran L. Liu Y. Zhong S. H. Zhou P. P. Liao M. X. (2018). Knockdown of hnRNP A2/B1 inhibits cell proliferation, invasion and cell cycle triggering apoptosis in cervical cancer via PI3K/AKT signaling pathway. *Oncol. Rep.* 39 939–950. 29328485 10.3892/or.2018.6195 PMC5802035

[B210] Shi Y. (2017). Mechanistic insights into precursor messenger RNA splicing by the spliceosome. *Nat. Rev. Mol. Cell Biol.* 18 655–670. 10.1038/nrm.2017.86 28951565

[B211] Shilo A. Siegfried Z. Karni R. (2015). The role of splicing factors in deregulation of alternative splicing during oncogenesis and tumor progression. *Mol. Cell Oncol.* 2:e970955. 10.4161/23723548.2014.970955 27308389 PMC4905244

[B212] Shin C. Feng Y. Manley J. L. (2004). Dephosphorylated SRp38 acts as a splicing repressor in response to heat shock. *Nature* 427 553–558. 10.1038/nature02288 14765198

[B213] Shiraishi Y. Kataoka K. Chiba K. Okada A. Kogure Y. Tanaka H. (2018). A comprehensive characterization of cis-acting splicing-associated variants in human cancer. *Genome Res.* 28 1111–1125. 10.1101/gr.231951.117 30012835 PMC6071634

[B214] Shultz J. C. Goehe R. W. Murudkar C. S. Wijesinghe D. S. Mayton E. K. Massiello A. (2011). SRSF1 regulates the alternative splicing of caspase 9 via a novel intronic splicing enhancer affecting the chemotherapeutic sensitivity of non-small cell lung cancer cells. *Mol. Cancer Res.* 9 889–900. 10.1158/1541-7786.mcr-11-0061 21622622 PMC3140550

[B215] Shultz J. C. Goehe R. W. Wijesinghe D. S. Murudkar C. Hawkins A. J. Shay J. W. (2010). Alternative splicing of caspase 9 is modulated by the phosphoinositide 3-kinase/Akt pathway via phosphorylation of SRp30a. *Cancer Res.* 70 9185–9196. 10.1158/0008-5472.can-10-1545 21045158 PMC3059118

[B216] Singh B. Eyras E. (2017). The role of alternative splicing in cancer. *Transcription* 8 91–98. 10.1080/21541264.2016.1268245 28005460 PMC5423477

[B217] Siomi H. Dreyfuss G. (1995). A nuclear localization domain in the hnRNP A1 protein. *J. Cell Biol.* 129 551–560. 10.1083/jcb.129.3.551 7730395 PMC2120450

[B218] Smith-Roe S. L. Nakamura J. Holley D. Chastain P. D. Rosson G. B. Simpson D. A. (2015). SWI/SNF complexes are required for full activation of the DNA-damage response. *Oncotarget* 6 732–745. 10.18632/oncotarget.2715 25544751 PMC4359251

[B219] Sokol E. Kedzierska H. Czubaty A. Rybicka B. Rodzik K. Tanski Z. (2018). microRNA-mediated regulation of splicing factors SRSF1, SRSF2 and hnRNP A1 in context of their alternatively spliced 3′UTRs. *Exp. Cell Res.* 363 208–217. 10.1016/j.yexcr.2018.01.009 29331391

[B220] Somberg M. Li X. Johansson C. Orru B. Chang R. Rush M. (2011). Serine/arginine-rich protein 30c activates human papillomavirus type 16 L1 mRNA expression via a bimodal mechanism. *J. Gen. Virol.* 92(Pt 10) 2411–2421. 10.1099/vir.0.033183-0 21697349

[B221] Somberg M. Schwartz S. (2010). Multiple ASF/SF2 sites in the human papillomavirus type 16 (HPV-16) E4-coding region promote splicing to the most commonly used 3′-splice site on the HPV-16 genome. *J. Virol.* 84 8219–8230. 10.1128/jvi.00462-10 20519389 PMC2916536

[B222] Somberg M. Zhao X. Frohlich M. Evander M. Schwartz S. (2008). Polypyrimidine tract binding protein induces human papillomavirus type 16 late gene expression by interfering with splicing inhibitory elements at the major late 5′ splice site, SD3632. *J. Virol.* 82 3665–3678. 10.1128/jvi.02140-07 18216120 PMC2268445

[B223] Song L. Wang L. Li Y. Xiong H. Wu J. Li J. (2010). Sam68 up-regulation correlates with, and its down-regulation inhibits, proliferation and tumourigenicity of breast cancer cells. *J. Pathol.* 222 227–237. 10.1002/path.2751 20662004

[B224] Sorlie T. Perou C. M. Tibshirani R. Aas T. Geisler S. Johnsen H. (2001). Gene expression patterns of breast carcinomas distinguish tumor subclasses with clinical implications. *Proc. Natl. Acad. Sci. U.S.A.* 98 10869–10874. 10.1073/pnas.191367098 11553815 PMC58566

[B225] Sterne-Weiler T. Sanford J. R. (2014). Exon identity crisis: disease-causing mutations that disrupt the splicing code. *Genome Biol.* 15:201. 10.1186/gb4150 24456648 PMC4053859

[B226] Stockley J. Markert E. Zhou Y. Robson C. N. Elliott D. J. Lindberg J. (2015). The RNA-binding protein Sam68 regulates expression and transcription function of the androgen receptor splice variant AR-V7. *Sci. Rep.* 5:13426. 10.1038/srep13426 PMC4550848 26310125

[B227] Stoilov P. Daoud R. Nayler O. Stamm S. (2004). Human tra2-beta1 autoregulates its protein concentration by influencing alternative splicing of its pre-mRNA. *Hum. Mol. Genet.* 13 509–524. 10.1093/hmg/ddh051 14709600

[B228] Straub E. Fertey J. Dreer M. Iftner T. Stubenrauch F. (2015). Characterization of the human papillomavirus 16 E8 promoter. *J. Virol.* 89 7304–7313. 10.1128/jvi.00616-15 25948744 PMC4473580

[B229] Sudarsanam P. Winston F. (2000). The Swi/Snf family nucleosome-remodeling complexes and transcriptional control. *Trends Genet.* 16 345–351. 10904263 10.1016/s0168-9525(00)02060-6

[B230] Suk F. M. Lin S. Y. Lin R. J. Hsine Y. H. Liao Y. J. Fang S. U. (2015). Bortezomib inhibits Burkitt’s lymphoma cell proliferation by downregulating sumoylated hnRNP K and c-Myc expression. *Oncotarget* 6 25988–26001. 10.18632/oncotarget.4620 26317903 PMC4694880

[B231] Sun H. Liu T. Zhu D. Dong X. Liu F. Liang X. (2017). HnRNPM and CD44s expression affects tumor aggressiveness and predicts poor prognosis in breast cancer with axillary lymph node metastases. *Genes Chromosomes Cancer* 56 598–607. 10.1002/gcc.22463 28393427

[B232] Sun X. Haider Ali M. S. S. Moran M. (2017). The role of interactions of long non-coding RNAs and heterogeneous nuclear ribonucleoproteins in regulating cellular functions. *Biochem. J.* 474 2925–2935. 10.1042/bcj20170280 28801479 PMC5553131

[B233] Sun Y. Luo M. Chang G. Ren W. Wu K. Li X. (2017). Phosphorylation of Ser6 in hnRNPA1 by S6K2 regulates glucose metabolism and cell growth in colorectal cancer. *Oncol. Lett.* 14 7323–7331. 29344170 10.3892/ol.2017.7085 PMC5755035

[B234] Sveen A. Kilpinen S. Ruusulehto A. Lothe R. A. Skotheim R. I. (2016). Aberrant RNA splicing in cancer; expression changes and driver mutations of splicing factor genes. *Oncogene* 35 2413–2427. 10.1038/onc.2015.318 26300000

[B235] Sweetser D. A. Peniket A. J. Haaland C. Blomberg A. A. Zhang Y. Zaidi S. T. (2005). Delineation of the minimal commonly deleted segment and identification of candidate tumor-suppressor genes in del(9q) acute myeloid leukemia. *Genes Chromosomes Cancer* 44 279–291. 10.1002/gcc.20236 16015647

[B236] Tacke R. Tohyama M. Ogawa S. Manley J. L. (1998). Human Tra2 proteins are sequence-specific activators of pre-mRNA splicing. *Cell* 93 139–148. 10.1016/s0092-8674(00)81153-8 9546399

[B237] Talukdar I. Sen S. Urbano R. Thompson J. Yates J. R. III Webster N. J. (2011). hnRNP A1 and hnRNP F modulate the alternative splicing of exon 11 of the insulin receptor gene. *PLoS One* 6:e27869. 10.1371/journal.pone.0027869 22132154 PMC3223206

[B238] Tang S. Tao M. McCoy J. P. Jr. Zheng Z. M. (2006). The E7 oncoprotein is translated from spliced E6^∗^I transcripts in high-risk human papillomavirus type 16- or type 18-positive cervical cancer cell lines via translation reinitiation. *J. Virol.* 80 4249–4263. 10.1128/jvi.80.9.4249-4263.2006 16611884 PMC1472016

[B239] Tang X. Kane V. D. Morre D. M. Morre D. J. (2011). hnRNP F directs formation of an exon 4 minus variant of tumor-associated NADH oxidase (ENOX2). *Mol. Cell Biochem.* 357 55–63. 10.1007/s11010-011-0875-5 21625959

[B240] Tarn W. Y. Steitz J. A. (1997). Pre-mRNA splicing: the discovery of a new spliceosome doubles the challenge. *Trends Biochem. Sci.* 22 132–137. 10.1016/s0968-0004(97)01018-9 9149533

[B241] Tauler J. Zudaire E. Liu H. Shih J. Mulshine J. L. (2010). hnRNP A2/B1 modulates epithelial-mesenchymal transition in lung cancer cell lines. *Cancer Res.* 70 7137–7147. 10.1158/0008-5472.can-10-0860 20807810

[B242] Taylor S. J. Anafi M. Pawson T. Shalloway D. (1995). Functional interaction between c-Src and its mitotic target, Sam 68. *J. Biol. Chem.* 270 10120–10124. 10.1074/jbc.270.17.10120 7537265

[B243] Tornesello M. L. Annunziata C. Tornesello A. L. Buonaguro L. Buonaguro F. M. (2018). Human oncoviruses and p53 tumor suppressor pathway deregulation at the origin of human cancers. *Cancers* 10:213. 10.3390/cancers10070213 29932446 PMC6071257

[B244] Tornesello M. L. Faraonio R. Buonaguro L. Annunziata C. Starita N. Cerasuolo A. (2020). The role of microRNAs, long non-coding RNAs, and circular RNAs in cervical cancer. *Front. Oncol.* 10:150. 10.3389/fonc.2020.00150 32154165 PMC7044410

[B245] Turunen J. J. Niemela E. H. Verma B. Frilander M. J. (2013). The significant other: splicing by the minor spliceosome. *Wiley Interdiscip. Rev. RNA* 4 61–76. 10.1002/wrna.1141 23074130 PMC3584512

[B246] Ule J. Blencowe B. J. (2019). Alternative splicing regulatory networks: functions, mechanisms, and evolution. *Mol. Cell* 76 329–345. 10.1016/j.molcel.2019.09.017 31626751

[B247] Urbanski L. M. Leclair N. Anczukow O. (2018). Alternative-splicing defects in cancer: splicing regulators and their downstream targets, guiding the way to novel cancer therapeutics. *Wiley Interdiscip. Rev. RNA* 9:e1476. 10.1002/wrna.1476 29693319 PMC6002934

[B248] van D. K. Li Z. Xirasagar S. Maes P. Kaminsky D. Liou D. (2017). The Papillomavirus Episteme: a major update to the papillomavirus sequence database. *Nucleic Acids Res.* 45 D499–D506. 28053164 10.1093/nar/gkw879 PMC5210616

[B249] Venables J. P. Bourgeois C. F. Dalgliesh C. Kister L. Stevenin J. Elliott D. J. (2005). Up-regulation of the ubiquitous alternative splicing factor Tra2beta causes inclusion of a germ cell-specific exon. *Hum. Mol. Genet.* 14 2289–2303. 10.1093/hmg/ddi233 16000324

[B250] Wahl M. C. Will C. L. Luhrmann R. (2009). The spliceosome: design principles of a dynamic RNP machine. *Cell* 136 701–718. 10.1016/j.cell.2009.02.009 19239890

[B251] Wan L. Yu W. Shen E. Sun W. Liu Y. Kong J. (2019). SRSF6-regulated alternative splicing that promotes tumour progression offers a therapy target for colorectal cancer. *Gut* 68 118–129. 10.1136/gutjnl-2017-314983 29114070

[B252] Wang B. D. Lee N. H. (2018). Aberrant RNA splicing in cancer and drug resistance. *Cancers* 10:458. 10.3390/cancers10110458 30463359 PMC6266310

[B253] Wang C. Norton J. T. Ghosh S. Kim J. Fushimi K. Wu J. Y. (2008). Polypyrimidine tract-binding protein (PTB) differentially affects malignancy in a cell line-dependent manner. *J. Biol. Chem.* 283 20277–20287. 10.1074/jbc.m803682200 18499661 PMC2459264

[B254] Wang F. Fu X. Chen P. Wu P. Fan X. Li N. (2017). SPSB1-mediated HnRNP A1 ubiquitylation regulates alternative splicing and cell migration in EGF signaling. *Cell Res.* 27 540–558. 10.1038/cr.2017.7 28084329 PMC5385621

[B255] Wang G. S. Cooper T. A. (2007). Splicing in disease: disruption of the splicing code and the decoding machinery. *Nat. Rev. Genet.* 8 749–761. 10.1038/nrg2164 17726481

[B256] Wang Y. Liu J. Huang B. O. Xu Y. M. Li J. Huang L. F. (2015). Mechanism of alternative splicing and its regulation. *Biomed. Rep.* 3 152–158. 25798239 10.3892/br.2014.407 PMC4360811

[B257] Wang Z. Burge C. B. (2008). Splicing regulation: from a parts list of regulatory elements to an integrated splicing code. *RNA* 14 802–813. 10.1261/rna.876308 18369186 PMC2327353

[B258] Watson I. R. Takahashi K. Futreal P. A. Chin L. (2013). Emerging patterns of somatic mutations in cancer. *Nat. Rev. Genet.* 14 703–718. 10.1038/nrg3539 24022702 PMC4014352

[B259] Will C. L. Luhrmann R. (2011). Spliceosome structure and function. *Cold Spring Harb. Perspect. Biol.* 3:a003707. 10.1101/cshperspect.a003707 PMC3119917 21441581

[B260] Williams V. M. Filippova M. Filippov V. Payne K. J. Duerksen-Hughes P. (2014). Human papillomavirus type 16 E6^∗^ induces oxidative stress and DNA damage. *J. Virol.* 88 6751–6761. 10.1128/jvi.03355-13 24696478 PMC4054338

[B261] Wu C. Kajitani N. Schwartz S. (2017). Splicing and polyadenylation of human papillomavirus type 16 mRNAs. *Int. J. Mol. Sci.* 18:366. 10.3390/ijms18020366 28208770 PMC5343901

[B262] Wu H. Sun S. Tu K. Gao Y. Xie B. Krainer A. R. (2010). A splicing-independent function of SF2/ASF in microRNA processing. *Mol. Cell* 38 67–77. 10.1016/j.molcel.2010.02.021 20385090 PMC3395997

[B263] Wu X. Yang Y. Huang Y. Chen Y. Wang T. Wu S. (2018). RNA-binding protein AUF1 suppresses miR-122 biogenesis by down-regulating Dicer1 in hepatocellular carcinoma. *Oncotarget* 9 14815–14827. 10.18632/oncotarget.24079 29599909 PMC5871080

[B264] Xiao J. Wang Q. Yang Q. Wang H. Qiang F. He S. (2018). Clinical significance and effect of Sam68 expression in gastric cancer. *Oncol. Lett.* 15 4745–4752. 29552114 10.3892/ol.2018.7930 PMC5840748

[B265] Xue Y. Bellanger S. Zhang W. Lim D. Low J. Lunny D. (2010). HPV16 E2 is an immediate early marker of viral infection, preceding E7 expression in precursor structures of cervical carcinoma. *Cancer Res.* 70 5316–5325. 10.1158/0008-5472.can-09-3789 20530671

[B266] Yang H. Zhu R. Zhao X. Liu L. Zhou Z. Zhao L. (2019). Sirtuin-mediated deacetylation of hnRNP A1 suppresses glycolysis and growth in hepatocellular carcinoma. *Oncogene* 38 4915–4931. 10.1038/s41388-019-0764-z 30858544

[B267] Yang S. Jia R. Bian Z. (2018). SRSF5 functions as a novel oncogenic splicing factor and is upregulated by oncogene SRSF3 in oral squamous cell carcinoma. *Biochim. Biophys. Acta Mol. Cell Res.* 1865 1161–1172. 10.1016/j.bbamcr.2018.05.017 29857020

[B268] Yeo-Teh N. S. L. Ito Y. Jha S. (2018). High-risk human papillomaviral oncogenes E6 and E7 target key cellular pathways to achieve oncogenesis. *Int. J. Mol. Sci.* 19:1706. 10.3390/ijms19061706 29890655 PMC6032416

[B269] Yoshida T. Kim J. H. Carver K. Su Y. Weremowicz S. Mulvey L. (2015). CLK2 is an oncogenic kinase and splicing regulator in breast cancer. *Cancer Res.* 75 1516–1526. 10.1158/0008-5472.can-14-2443 25670169

[B270] Yu C. Guo J. Liu Y. Jia J. Jia R. Fan M. (2015). Oral squamous cancer cell exploits hnRNP A1 to regulate cell cycle and proliferation. *J. Cell Physiol.* 230 2252–2261. 10.1002/jcp.24956 25752295

[B271] Yu H. Gong L. Wu C. Nilsson K. Li-Wang X. Schwartz S. (2018). hnRNP G prevents inclusion on the HPV16 L1 mRNAs of the central exon between splice sites SA3358 and SD3632. *J. Gen. Virol.* 99 328–343. 10.1099/jgv.0.001019 29458523

[B272] Zhang Q. Di C. Yan J. Wang F. Qu T. Wang Y. (2019a). Inhibition of SF3b1 by pladienolide B evokes cycle arrest, apoptosis induction and p73 splicing in human cervical carcinoma cells. *Artif Cells Nanomed. Biotechnol.* 47 1273–1280. 10.1080/21691401.2019.1596922 30963795

[B273] Zhang Q. Lv R. Guo W. Li X. (2019b). microRNA-802 inhibits cell proliferation and induces apoptosis in human cervical cancer by targeting serine/arginine-rich splicing factor 9. *J. Cell Biochem.* 120 10370–10379. 10.1002/jcb.28321 30565744

[B274] Zhang T. Wan C. Shi W. Xu J. Fan H. Zhang S. (2015). The RNA-binding protein Sam68 regulates tumor cell viability and hepatic carcinogenesis by inhibiting the transcriptional activity of FOXOs. *J. Mol. Histol.* 46 485–497. 10.1007/s10735-015-9639-y 26438629

[B275] Zhang Y. Wu D. Wang D. (2020). Long non-coding RNA ARAP1-AS1 promotes tumorigenesis and metastasis through facilitating proto-oncogene c-Myc translation via dissociating PSF/PTB dimer in cervical cancer. *Cancer Med.* 9 1855–1866. 10.1002/cam4.2860 31953923 PMC7050100

[B276] Zhang Y. Yan L. Zeng J. Zhou H. Liu H. Yu G. (2019). Pan-cancer analysis of clinical relevance of alternative splicing events in 31 human cancers. *Oncogene* 38 6678–6695. 10.1038/s41388-019-0910-7 31391553

[B277] Zhang Z. Li J. Zheng H. Yu C. Chen J. Liu Z. (2009). Expression and cytoplasmic localization of SAM68 is a significant and independent prognostic marker for renal cell carcinoma. *Cancer Epidemiol. Biomarkers Prev.* 18 2685–2693. 10.1158/1055-9965.epi-09-0097 19755649

[B278] Zhang Z. Xu Y. Sun N. Zhang M. Xie J. Jiang Z. (2014). High Sam68 expression predicts poor prognosis in non-small cell lung cancer. *Clin. Transl. Oncol.* 16 886–891. 10.1007/s12094-014-1160-3 24522888

[B279] Zhao X. Fay J. Lambkin H. Schwartz S. (2007). Identification of a 17-nucleotide splicing enhancer in HPV-16 L1 that counteracts the effect of multiple hnRNP A1-binding splicing silencers. *Virology* 369 351–363. 10.1016/j.virol.2007.08.002 17869320

[B280] Zhao X. Oberg D. Rush M. Fay J. Lambkin H. Schwartz S. (2005). A 57-nucleotide upstream early polyadenylation element in human papillomavirus type 16 interacts with hFip1, CstF-64, hnRNP C1/C2, and polypyrimidine tract binding protein. *J. Virol.* 79 4270–4288. 10.1128/jvi.79.7.4270-4288.2005 15767428 PMC1061554

[B281] Zhao X. Rush M. Schwartz S. (2004). Identification of an hnRNP A1-dependent splicing silencer in the human papillomavirus type 16 L1 coding region that prevents premature expression of the late L1 gene. *J. Virol.* 78 10888–10905. 10.1128/jvi.78.20.10888-10905.2004 15452209 PMC521837

[B282] Zhao X. Schwartz S. (2008). Inhibition of HPV-16 L1 expression from L1 cDNAs correlates with the presence of hnRNP A1 binding sites in the L1 coding region. *Virus Genes* 36 45–53. 10.1007/s11262-007-0174-0 18040766

[B283] Zheng Z. M. Baker C. C. (2006). Papillomavirus genome structure, expression, and post-transcriptional regulation. *Front. Biosci.* 11:2286–2302. 10.2741/1971 16720315 PMC1472295

[B284] Zheng Z. M. Tao M. Yamanegi K. Bodaghi S. Xiao W. (2004). Splicing of a cap-proximal human Papillomavirus 16 E6E7 intron promotes E7 expression, but can be restrained by distance of the intron from its RNA 5′ cap. *J. Mol. Biol.* 337 1091–1108. 10.1016/j.jmb.2004.02.023 15046980

[B285] Zheng Z. Z. Sun Y. Y. Zhao M. Huang H. Zhang J. Xia N. S. (2013). Specific interaction between hnRNP H and HPV16 L1 proteins: implications for late gene auto-regulation enabling rapid viral capsid protein production. *Biochem. Biophys. Res. Commun.* 430 1047–1053. 10.1016/j.bbrc.2012.12.042 23261416

[B286] Zhou X. Li Q. He J. Zhong L. Shu F. Xing R. (2017). HnRNP-L promotes prostate cancer progression by enhancing cell cycling and inhibiting apoptosis. *Oncotarget* 8 19342–19353. 10.18632/oncotarget.14258 28038443 PMC5386688

[B287] Zhou X. Li X. Cheng Y. Wu W. Xie Z. Xi Q. (2014). BCLAF1 and its splicing regulator SRSF10 regulate the tumorigenic potential of colon cancer cells. *Nat. Commun.* 5:4581. 10.1038/ncomms5581 25091051

[B288] Zhou X. Li X. Yu L. Wang R. Hua D. Shi C. (2019). The RNA-binding protein SRSF1 is a key cell cycle regulator via stabilizing NEAT1 in glioma. *Int. J. Biochem. Cell Biol.* 113 75–86. 10.1016/j.biocel.2019.06.003 31200124

[B289] Zhou Z. Fu X. D. (2013). Regulation of splicing by SR proteins and SR protein-specific kinases. *Chromosoma* 122 191–207. 10.1007/s00412-013-0407-z 23525660 PMC3660409

[B290] Zhou Z. J. Dai Z. Zhou S. L. Fu X. T. Zhao Y. M. Shi Y. H. (2013). Overexpression of HnRNP A1 promotes tumor invasion through regulating CD44v6 and indicates poor prognosis for hepatocellular carcinoma. *Int. J. Cancer* 132 1080–1089. 10.1002/ijc.27742 22821376

[B291] Zhu H. Zheng T. Yu J. Zhou L. Wang L. (2018). LncRNA XIST accelerates cervical cancer progression via upregulating Fus through competitively binding with miR-200a. *Biomed. Pharmacother.* 105 789–797. 10.1016/j.biopha.2018.05.053 29909347

